# IKKβ deletion from CNS macrophages increases neuronal excitability and accelerates the onset of EAE, while from peripheral macrophages reduces disease severity

**DOI:** 10.1186/s12974-024-03023-9

**Published:** 2024-01-27

**Authors:** Maria Avloniti, Maria Evangelidou, Maria Gomini, Theodore Loupis, Mary Emmanouil, Adamantia Mitropoulou, Theodore Tselios, Hans Lassmann, Agnès Gruart, José M. Delgado-García, Lesley Probert, Vasiliki Kyrargyri

**Affiliations:** 1 Laboratory of Molecular Genetics, Hellenic Pasteur Institute, Athens, Greece; 2 Greek Genome Centre, Biomedical Research Foundation of the Academy of Athens (BRFAA), Athens, Greece; 3 Haematology Research Laboratory, Biomedical Research Foundation of the Academy of Athens (BRFAA), Athens, Greece; 4 Department of Chemistry, University of Patras, Patras, Greece; 5 Department of Neuroimmunology, Centre for Brain Research, Medical University of Vienna, Vienna, Austria; 6 Division of Neurosciences, Pablo de Olavide University, 41013 Seville, Spain

**Keywords:** Microglia, NF-κB, Multiple sclerosis, In vivo electrophysiology, LTP

## Abstract

**Background:**

Multiple sclerosis (MS) is a neuroinflammatory demyelinating disease characterized by motor deficits and cognitive decline. Many immune aspects of the disease are understood through studies in the experimental autoimmune encephalomyelitis (EAE) model, including the contribution of the NF-κB transcription factor to neuroinflammation. However, the cell-specific roles of NF-κB to EAE and its cognitive comorbidities still needs further investigation. We have previously shown that the myeloid cell NF-κB plays a role in the healthy brain by exerting homeostatic regulation of neuronal excitability and synaptic plasticity and here we investigated its role in EAE.

**Methods:**

We used constitutive MφIKKβΚΟ mice, in which depletion of IKKβ, the main activating kinase of NF-κB, was global to CNS and peripheral macrophages, and ΜgΙΚΚβKO mice, in which depletion was inducible and specific to CNS macrophages by 28 days after tamoxifen administration. We subjected these mice to MOG_35-55_ induced EAE and cuprizone-induced demyelination. We measured pathology by immunohistochemistry, investigated molecular mechanisms by RNA sequencing analysis and studied neuronal functions by in vivo electrophysiology in awake animals.

**Results:**

Global depletion of IKKβ from myeloid cells in MφIKKβΚΟ mice accelerated the onset and significantly supressed chronic EAE. Knocking out IKKβ only from CNS resident macrophages accelerated the onset and exacerbated chronic EAE, accompanied by earlier demyelination and immune cell infiltration but had no effect in cuprizone-induced demyelination. Peripheral T cell effector functions were not affected by myeloid cell deletion of IKKβ, but CNS resident mechanisms, such as microglial activation and neuronal hyperexcitability were altered from early in EAE. Lastly, depletion of myeloid cell IKKβ resulted in enhanced late long-term potentiation in EAE.

**Conclusions:**

IKKβ-mediated activation of NF-κΒ in myeloid cells has opposing roles in EAE depending on the cell type and the disease stage. In CNS macrophages it is protective while in peripheral macrophages it is disease-promoting and acts mainly during chronic disease. Although clinically protective, CNS myeloid cell IKKβ deletion dysregulates neuronal excitability and synaptic plasticity in EAE. These effects of IKKβ on brain cognitive abilities deserve special consideration when therapeutic interventions that inhibit NF-κB are used in MS.

**Supplementary Information:**

The online version contains supplementary material available at 10.1186/s12974-024-03023-9.

## Introduction

MS is a progressive neuroinflammatory demyelinating (autoimmune) disease of the CNS that is characterized by motor, sensory and cognitive decline. MS patients mainly suffer from motor paralysis, which is consequently the main target of the approved drugs for MS so far. However, cognitive decline is also significant, with learning and memory, information processing speed, attention and visual–spatial abilities shown to be affected in 40–70% of MS patients [1, 2]. The autoimmune part of the disease can be modeled in animals by experimental autoimmune encephalomyelitis (EAE), which induces gradual paralysis in response to an immunization with peptides matching highly immunogenic regions of myelin proteins, such as myelin oligodendrocyte protein (MOG). EAE is a T cell-mediated model, where T cells are activated by MOG in the periphery and then transmigrate into the CNS where they initiate a neuroinflammatory cascade of events that involves among others, production of chemokines, attraction of other immune cells into the CNS and activation of the resident macrophages, that ultimately lead to neurodegeneration, axonal damage and demyelination. Many aspects of the immune part of the disease are already known by previous studies [3, 4], however the involvement of CNS macrophages to disease and its cognitive comorbidities still need further investigation.

NF-κB is a transcription factor that is expressed by all cells and has multiple roles in health and disease. In most resting cells, NF-κB is sequestered in the cytoplasm through interacting with any of a family of inhibitors of κB (IκB) proteins, such as IκBa, IκBb, and p100. Upon stimulated signals, IκB kinase (IKK) (mainly IKKβ) rapidly phosphorylates IκB and facilitates its ubiquitination and proteasomal degradation, which ultimately enables the entrance of NF-κB into the nucleus and elicits its transcriptional activity. Given that NF-κB affects almost the entire arsenal of immune guardians and immune cells [5], special concern has gradually been focused on the pivotal role of NF-κB dysregulation in many autoimmune inflammatory diseases. In MS, pathological studies have detected activated NF-κB subunits in macrophages, microglia, oligodendrocytes, astrocytes, neurons, perivascular lymphocytes near or in active MS plaques [6], however the impact of its activation on the different cell types to pathology and its cognitive comorbidities still needs further investigation. We have previously shown that the myeloid NF-κB signaling play a role in the healthy brain by exerting homeostatic regulation of neuronal excitability, synaptic plasticity, and behavior in the healthy brain [7]. Here we investigated the role of myeloid NF-κB signaling in CNS pathology, particularly in EAE. We investigated the differential effects of NF-κB activity in CNS versus peripheral macrophages in EAE and whether depleting myeloid NF-κB activity may affect neuronal excitability and synaptic plasticity in this model, which is the molecular basis of cognitive functions, such as learning and memory.

Using transgenic animals, immunohistochemistry, RNA sequencing and in vivo electrophysiology in awake animals, we show that the IKKβ-NF-κΒ activity of myeloid cells has different roles in EAE depending on the cell type and the stage of the disease. IKKβ expressed in CNS macrophages protected against CNS infiltration by immune cells while that expressed in peripheral macrophages enhanced disease activity, particularly during the chronic phase of the disease. Peripheral antigen-specific T cell responses were not affected by myeloid IKKβ deletion, but CNS resident mechanisms, such as microglial activation and neuronal hyperexcitability were observed at the very early stages of EAE, suggesting that alterations in the homeostatic regulation of microglia–neuron communication at these early time points of EAE may accelerate the onset of the disease and the overall pathology in these mice. Lastly, depletion of myeloid IKKβ resulted in enhanced late long-term potentiation in EAE, suggesting that brain cognitive abilities may be also affected by the use of approved treatments against NF-κB activity in MS, which needs to be taken into consideration.

## Materials and methods

### Mice

Adult (2–4 months) female and male mice were used in this study. Mice containing a conditional IKKβ allele in which exon 3 of the *Ikbkb* gene, encoding the IKKβ activation loop, is flanked by loxP sites (IKKβF/F) were donated by M. Karin (UCSD) and have been described previously [8, 9]. Mice with a constitutive depletion of IKKβ in cells of myeloid origin (MφIKKβKO) were generated by crossing IKKβF/F with transgenic mice that express a CD11b promoter-driven Cre recombinase (CD11b-Cre) and have been described previously [7]. Mice with tamoxifen-inducible ablation of the *Ikbkb* gene were generated by crossing Cx3cr1Cre^ER/EYFP+/−^ (Jackson Lab, 021160) and IKKβF/F adult mice. The Cre recombinase in these mice was induced by 2 or 4 (as indicated) daily applications of tamoxifen (Sigma; T5648), which was prepared as stock of 20 mg/ml by dissolving the powder in a solution containing 90% corn oil and 10% EtOH and was administered at 120 mg/kg by oral gavage. The Rosa26^tdTomato+/−^ mice (Jackson Lab, 007914) were used as a reporter strain for Cre recombination efficacy. For Fig. 5A–C, adult female C57BL/6 mice from the Animal Facility of the Hellenic Pasteur Institute (HPI) were used. For all experiments, mice with a C57BL/6 background (backcrossed at least 12 times) were used, and experimental and control mice were age-matched and usually littermates. The maintenance of laboratory mice was performed in SPF (Specific Pathogen Free) conditions at the approved establishments of Department of Animal Models for Biomedical Research, HPI under the registered codes EL25BIO011, EL25BIO012 and EL25BIO013.

Animals were housed at room temperature 22 ± 2 °C, relative humidity 40–70% with a 12 h light/12 h dark cycle. All procedures complied to PD 56/2013 and European Directive 2010/63/EU, welfare and ethical use of laboratory animals based on 3 + 1R: Replacement, Reduction, Refinement and Respect and the guidelines of PREPARE (Planning Research and Experimental Procedures on Animals: Recommendations for Excellence), and ARRIVE (Animal Research: Reporting in vivo experiments). The experimental protocol has been positively evaluated by the Institutional Protocol Evaluation Committee and was licensed under the registered codes 2581/31-05-2018 and 990461/22–22-2023, by the Official Veterinary Authorities of the Attiki Prefecture. Animal welfare was assessed by qualified staff and supervised daily by an in-house veterinarian.

### Gene targeting analysis of the tamoxifen-inducible MgIKKβKO system

The specificity of gene targeting in the MgIKKβKO system was evaluated by crossing tamoxifen-inducible Cx3cr1-Cre^ER/EYFP+/−^ mice, which constitutively express EYFP in CX3CR1^+^ cells, with Rosa^26tdTomato+/−^ reporter mice, as previously described [10, 11]. At 5 and 28 days after tamoxifen administration, mononuclear cells isolated from the spleen, spinal cord, and brain of Cx3cr1-Cre^ER+/−^Rosa^26tdTomato+/−^ mice were labeled for the pan-macrophage marker CD11b and the recombination efficacy was measured by FACS analysis as the percentage of EYFP^+^CD11b^+^ cells that were also positive for tdTomato. (Additional file 4: Fig. S4). In splenocytes the detected recombination dropped from almost 50% at 5 days after tamoxifen to ~ 5% at 28 days after tamoxifen (Additional file 4: Fig. S4B &C). In contrast, in both CNS tissues, brain and spinal cord, the recombination was maintained to almost 90% at all time points tested (Additional file 4: Fig. S4B, C).

We next performed immunohistochemistry on isolated CNS and peripheral tissues from MgIKKβKO and IKKβF/F control mice, that had been treated with tamoxifen for 5 and 28 days, to label, quantify and compare the ΙΚKβ protein levels between them. We used the anti-IKKβ antbibody (IKKβ (L570) antibody, cell signaling, #2678) in paraffin and cryostat sections at different concentrations and with different antigen retrieval approaches, such as EDTA and citrate buffer, but the results were not conclusive due to technical reasons, such as signal background and non-specific staining. We thus, additionally used a different experimental approach to test whether the depletion of IKKβ in MgIKKβKO mice affected the NF-κB activation pathway at a functional level in vitro. We isolated peritoneal macrophages from IKKβF/F and MgIKKβKO mice at 5 days after tamoxifen, and stimulated them in vitro for 24 h with LPS, a known activator of the NF-κB pathway. We then measured the percentage of CD11b^+^ cells that produced the inflammatory cytokine TNF, a known NF-κB target, by FACS. To examine whether the in vivo application of tamoxifen might affect the response of the primary isolated peritoneal macrophages to LPS, we also added a control group of mice, IKKβF/F and MgIKKβKO, that had not been treated with tamoxifen but injected only with corn oil diluted in ethanol (IKKβF/F no TAM and MgIKKβΚΟ no TAM). The analysis showed that the tamoxifen treatment did not affect the response of the cells to LPS. Furthermore, the proportion (%) of CD11b^+^ that produced TNF in the MgIKKβΚΟ peritoneal macrophages treated with LPS was lower compared to IKKβF/F with or without TAM and even lower compared to MgIKKβΚΟ no TAM group (Additional file 5: Fig. S5A and B). These data indicate that the depletion of IKKβ in the tamoxifen-inducible MgIKKβKO system affects the activation of the NF-κB signaling pathway and reduces the production of downstream inflammatory targets.

### EAE induction and evaluation

EAE was induced by subcutaneous tail base injections of 39 μg of rat MOG_35-55_ dissolved in 100μL saline and emulsified in 100μL of complete Freund’s adjuvant (CFA) (Sigma-Aldrich) as previously described [12]. CFA was supplemented with 400 μg/injection of H37Ra Mycobacterium tuberculosis (Sigma-Aldrich). Mice also received intraperitoneal injection of 200 ng of Bordetella pertussis toxin (PTx) (Difco) at the time of immunization (day 0) and 48 h later (day 2). Mice were monitored daily and assessed for clinical signs of disease according to the following scoring system, which was based on [13]: grade 0, no clinical symptom; grade 1, limp tail; grade 2, hindlimb weakness; grade 3, hindlimb paralysis; grade 4, forelimb and hindlimb paralysis; grade 5 moribund or dead (0.5 graduations represent intermediate scores). Animals with a score of 4 or above were euthanized. All mice were allowed free access to food and water throughout the experiments.

### Cuprizone model of demyelination and remyelination

Demyelination was induced by feeding 8- to 10-week-old male mice (28—35 gr) a powdered standard mouse chow containing 0.2% w/w CPZ (Sigma-Aldrich; catalogue C9012), as previously described [14]. Cuprizone feeding was maintained for 6 weeks, and thereafter mice were put on a normal chow for another 2 weeks. The control (naive) mice were fed a powdered standard mouse chow but without CPZ. Mice were killed at different time points to cover the progression of CPZ-induced demyelination (CPZ 3 and 5 weeks) and remyelination (CPZ 6 + 2 weeks) and fixed by transcardial perfusion with 4% paraformaldehyde (PFA) in ice-cold PBS, at killing by carbon dioxide inhalation. Brains were then removed and further fixed for 24 h in 4% PFA. Then, the left hemispheres were embedded in paraffin for sectioning and DAB immunohistochemistry. All experimental mice were weighed at the day of CPZ feeding initiation and once per week until killing.

### Histopathological analysis

Mice were transcardially perfused with ice-cold 4% PFA at killing by carbon dioxide inhalation. For EAE experiments, the vertebral column was dissected and post-fixed in the same fixative for 48 h at 4 °C, spinal cords were removed, embedded in paraffin and processed for standard histopathological analysis. Comparative analyses were made in the lumbar spinal cord. For cuprizone experiments, brains were removed, post-fixed in the same fixative overnight at 4 °C, embedded in paraffin and serial coronal paraffin sections (5 μM) were cut through the corpus callosum corresponding to Sidman sections 295–305. Comparative analyses were made in the midline corpus callosum, as previously described [14]. CNS infiltration was visualized by haematoxylin and eosin (H&E), demyelination by Luxol fast blue (LFB) staining, axon damage by Bielschowsky’s silver stain. Peroxidase/DAB immunohistochemistry was performed using a standard methodology, as previously described [14, 15]. In brief, paraffin sections were initially de-paraffinized in xylene and re-hydrated in a descending ethanol series (100%, 96%, 70%, 50%) and dH_2_O. Endogenous peroxidase activity was blocked by incubation of the sections in methanol/0.2% H_2_O_2_ for 30 min. Antigen retrieval was performed in a food steamer with 10 mM Tris/1 mM EDTA buffer (pH 8.5) for 20 min. The slices were then washed in PBS, blocked in 10% FBS in PBS for 1 h at room temperature and incubated in the following primary antibodies, rabbit anti-Iba1 (1/500; Wako chemicals; catalogue 019–19741), rabbit anti-GFAP (1/300; Dako; catalogue Z0334), rabbit anti-CD3 (1/2000; Neomarkers; RM-9107), anti-Mac3 (1/200; clone M3/84; Serotec), rabbit anti-rat iNOS (1/375; Chemicon; AB1631) or polyclonal rabbit anti-p22phox (1/100; Santa Cruz Biotexh; sc-20781), overnight at 4 °C. Sections were washed in PBS and incubated with biotinylated anti-rabbit or anti-mouse or anti-rat secondary antibody (1:1000 in blocking buffer, Vector Laboratories) for 1 h, at room temperature. After washing in PBS, an avidin–biotin based peroxidase complex (PK-6100, vector Laboratories) was used for detection of the biotinylated antibodies and immune complexes were visualized by monitored incubation of the slices in 3,3’-diaminobenzidine tetrachloride (DAB) (PanReac Applichem, A0596) in the presence of 10% H_2_O_2_.

Tissue sections were viewed with an Olympus BX-50 microscope and images captured with an Olympus DP71 microscope digital camera using cell^A imaging software (Soft Imaging System GmbH). Quantitative histopathological analysis was performed using Image J software, as previously described [14]. CNS infiltration was determined by semi-quantitative scoring as follows: (1) foci of subarachnoid cell infiltration or meningeal inflammation; (2) diffuse subarachnoid infiltration or perivascular infiltration; (3) foci of parenchymal infiltration; (4) diffuse widespread parenchymal infiltration. Demyelination was evaluated by semi-quantitative scoring as follows; (0.5): single perivascular sleeves of demyelination, (1) ubiquitous perivascular or sub-pial demyelination; (2) confluent demyelinated plaques; (3) profound focal demyelination, involving about 1/2 of the spinal cord white matter at least in one spinal cord segment and; (4) extensive demyelination, for instance complete demyelination of spinal cord white matter in one or more segment of cord. Images were scored blindly by two independent observers. Microgliosis and astrogliosis in the corpus callosum area were quantified by measuring the Iba1 and GFAP immunoreactivity, respectively, using Image J (mean grey value).

### Fluorescent immunohistochemistry

Mice were transcardially perfused with ice-cold 4% PFA at killing by carbon dioxide inhalation. To examine the morphology of parenchymal microglia, fixed spinal cords were equilibrated in 15% sucrose in PBS overnight at 4 °C and the next day in 30% sucrose in PBS overnight at 4 °C. Tissues were then embedded in Optimal Cutting Temperature Compound (Tissue-Tek OCT Compound, Sakura) and serial sagittal sections (50 μm) were cut on a cryostat (Leica CM3050 S). For fluorescent immunohistochemistry, sections were washed 3 times for 20 min each, in PBS and then blocked and permeabilized in 10% FBS and 0.5% Triton X-100 in PBS overnight, on a shaker at 4 °C. To label parenchymal microglia, slices were embedded at 4 °C for 48 h on a shaker in rabbit, anti-Iba1 primary antibody (1/1000, Wako chemicals, 019–19741), prepared in blocking buffer (10% FBS, 0.5% Triton in PBS). After washing 3 times for 20 min each in PBS, sections were incubated at 4 °C for 48 h on a shaker in an anti-rabbit secondary antibody (1/2000, goat, Alexa Fluor 568). Slices were then washed in PBS, incubated in DAPI (Invitrogen) and mounted on glass slides using Mowiol fluorescence mounting medium (10% Mowiol (Sigma), 25% Glycerol, 10% Tris 0.2 M pH = 8.5 in dH2O). To acquire three-dimensional (3D) fluorescent images, a Leica TCS SP8 confocal microscope was used. A 40X (NA 1.3) oil immersion objective was used for the images which were acquired as stacks of ~ 30 slices imaged at 1.5 μm z-stack step size. Images were typically 1024 by 1024 pixels and covered a square field of view 290.91 to 290.91 μm wide. These stacks were then merged together automatically to form the final image.

For CD45 immunofluorescence staining in whole mount spinal cord sections, we followed a protocol described previously [16]. In brief, the vertebral column was post-fixed and decalcified with 10% EDTA for 10 days at RT, equilibrated with 15% and 30% sucrose overnight each, mounted in OCT and snap frozen in melting isopentane. Transverse 40-mm cryostat sections were mounted on microscope slides for immunofluorescence staining. Sections were incubated with PBS containing 10% FCS and 0.5% Triton X-100 (Sigma- Aldrich) for 1 h at RT to block non-specific staining, followed by the primary antibody rat anti-CD45 mAb (1:200, 30-F11, Biolegend), overnight at 4 °C. The section were then incubated with the secondary antibody AlexaFluor-488 anti-rat IgG (1:1000, Invitrogen; A11006) for 1 h at RT. Nuclei were counterstained with DAPI (Invitrogen; catalog D1306). For the acquisition of the CD45 positive fluorescent images from the spinal cord segments the tile scan mode of the Leica TCS SP8 confocal microscope was used with a 20 × objective.

### Confocal image analysis

Confocal images were analyzed using a method based on MicroApp, a semi-automated and microglia cell-based analysis tool, which was designed to study the spatiotemporal changes of parenchymal CNS macrophages, particularly microglia which have a unique ramified morphology compared to the other CNS macrophages, in CNS demyelination models, as previously described [15]. It was performed using custom-written ImageJ2/Fiji, Matlab (Mathworks, version2018b) Java (Java SE8, Oracle Corporation) and Python scripts.

The first stage of analysis concerned raw data pre-processing to remove background, reduce noise and categorize the data according to disease time point into separate folders. A Python script was used to randomize each image so that the experimenter could be blinded to each experimental condition. Individual microglial cell isolation was performed manually, using Fiji/ImageJ, with the experimenter extracting only those cells whose entire cell body and fine processes were included into the 3D stack. At the second step of the procedure, 3D reconstruction of individual microglial cells was performed using the Imaris software, particularly the image analysis tool (v.9.2.1, Bitplane), and the Filament Tracer plugin, which uses an algorithm to reconstruct ramified cells in 3D. Briefly, the algorithm locates a sphere (cell body) as the beginning point and reconstructs the processes as either main or secondary branches. The tracing algorithm was applied to the isolated cells after setting manually a standard thresholding level for each cell, and the cell bodies were located after setting a 10 μm sphere as the beginning point, which reflects the average, across all time points, microglial cell body diameter (9.867 μm ± 0.22), measured for each cell as the maximum distance between two opposite edges of the soma.

For each individual cell, the Imaris software provided an output file in an excel form with the detailed arithmetic report, which was then used as an input to the third and final stage of analysis, which was the collection of all data per cell in one excel and calculations of the averages, standard deviation and error bars for each cell parameter, which was performed using custom-written script in Python. The microglial morphological properties analyzed here were the total length (in μm), which is the sum of the length of every process (main and secondary branches) per cell, the total volume (in μm3), which is the sum of the volume of every process (main and secondary branches) and the hypertrophic index, which is a ratio of the mean volume/total length.

### Flow cytometry

Draining lymph node cells, splenocytes, and spinal cord mononuclear cells were isolated from mice at the onset of EAE (Additional file 6: Fig. S6A). Mononuclear cells from the spinal cords were isolated by Percoll gradient centrifugation as previously described [16]. For detection of cell surface markers cells were fixed in 2% paraformaldehyde (PFA) solution in PBS for 20 min at 4 °C and immune-stained using fluorochrome-conjugated antibodies to CD11b APC (1/450, BD Pharmingen, Cat 553,312), CD4 PerCp-Cy5.5 (1/125, Biolegend Cat 103,131) and CD4 APC (1/300, BD Pharmingen, Cat 553,051) for 30 min at 4 °C. For intracellular staining of cytokines, cells where treated with phorbol 12-myristate 13-acetate (PMA) (10 ng/ml, Sigma-Aldrich) and ionomycin (1 mg/ml, Sigma-Aldrich) in the presence of brefeldin-A (5 mg/ml, Sigma-Aldrich) for 3 h at 37 °C/5% CO2, fixed with 2% PFA as above, permeabilized with 0.5% wt/vol saponin, and stained with fluorochrome-conjugated antibodies to IFNγ PE (1/200, BD Pharmingen, Cat 554,412), IL-17A APC (1/100, Biolegend, Cat 506,916), GM-CSF PE (1/100, BD Pharmingen, Cat 554,406), CD44 APC (1/300, BD Pharmingen, Cat 559,250), TNFa PE (1/150, BD Pharmingen, Cat 554,419), IL-10 PE (1/100, BD Pharmingen, Cat 554,467), and CD25 APC (1/200, Biolegend Cat 102,011). The Tregs were stained either by using a Mouse Regulatory T cell staining kit for FoxP3 (Cat 88–8111-40, eBioscience) or by triple staining with CD4 PerCP (1/125), IL-10 PE (1/100) and CD25 APC (1/200). Data were acquired using a FACSCalibur cytometer and analyzed with FlowJo software (Tree Star, Inc).

### Ιsolation and LPS stimulation of peritoneal macrophages

Peritoneal macrophages were harvested by peritoneal lavage from adult MgIKKβKO and ΙΚΚβF/F mice that had received 1 mL of 4% thioglycollate medium (Difco, Cat # 225,650, Becton Dickinson) i.p. 3 days previously. Cells were washed once in RPMI containing 5% FBS and allowed to adhere to 12-well plates at a concentration of 1 × 10^6^ cells/mL at 37 °C in a 5% CO2 incubator for 4 h. Then, the conditioned medium containing non-adherent cells was removed, replaced with new and the cells were incubated for another 16 h at 37 °C in a 5% CO2 incubator. The cells were then left untreated or treated with LPS (1 mg/ml, Sigma) for total 24 h. At 6 h Brefeldin A (5 μg/ml) was added to block the cytokine release. The cells were collected 24 h after LPS stimulation for FACS analysis.

### Bone marrow chimeras

Bone marrow cells were isolated from the femur of 2–4 months old ΜφIKKβΚΟ and IKKβF/F donor female mice. The cells were collected in ice-cold RPMI, centrifuged at 1200 rpm for 10 min at 4 °C and washed in PBS 1X. The pellet was then diluted in Gey’s erythrocyte lysis buffer for 4 min and further diluted in 15 mL RPMI to end the reaction. The cells were then filtered (0.2-μm pore sterile filters) and centrifuged at 1200 rpm for 10 min at 4 °C. The pellet was then diluted in 10 μL RPMI and the total number of cells per sample was measured in Neubauer. 6 × 10^6^ donor BM cells (in total 200 μL) were then injected into the lateral tail veins of the recipient mice, which had been exposed to full-body gamma irradiation, total 7.32 Gy in two cycles (3.66 Gy each cycle) with an interval of 3 h. The recipient mice were placed in filtered cages and left to recover for 6–8 weeks before EAE induction.

### T cell priming and proliferation assay

For [3H]-thymidine incorporation, splenocytes and draining lymph node (DLN) cells were recovered from mice immunized with MOG, as for EAE, or OVA, and cultured for 120 h in RPMI 1640 (Sigma-Aldrich) supplemented with 10% heat-inactivated FBS, 50 mM 2-mercaptoethanol (Sigma-Aldrich) and increasing concentrations of MOG or ovalbumin protein (OVA), respectively, as previously described [17]. Cells were stimulated with MOG or OVA in triplicate at 1 × 10^6^ cells/ml in round-bottom 96- well plates (Costar), pulsed with 1 mCi/5 × 105 cells [3H]-thymidine (Amersham Radiochemicals) for the last 16 h of culture. Control cells were stimulated in duplicates with medium alone as negative control. [3H]-thymidine incorporation was measured using a b scintillation counter. Results are expressed as radioactivity cpm, or the stimulation index (SI) calculated from the cpm of cells cultured in the presence of peptide divided by cpm of cells cultured in medium alone.

### RNA isolation and quantitative RT-PCR

Total RNA was extracted from ice-cold PBS-perfused, whole-brain tissues using TRIzol (Invitrogen, Paisley, UK) according to the manufacturer's instructions. DNAse-treated RNA samples were analyzed by quantitative RT-PCR using QuantiFast™ SYBRVR green RT PCR kit (Qiagen Inc.) according to the manufacturer's instructions. All reactions were performed using a LightCycler (Roche, Mannheim, Germany). At the end of each PCR run, melting curve analysis was performed to verify the integrity and homogeneity of PCR products. Gene expression levels were calculated using standard curves for each gene, which were created by plotting threshold cycle (CT) values versus the logarithm of serial diluted RNA concentrations. A least-square method was used for the determination of A and B values in the equation CT = A*Log (CRNA) + B. The coefficient of determination (R2) was greater than 0.99. Values were normalized using the respective values for the housekeeping gene, Gapdh. All results were analyzed using the LightCycler software version 3.5 (Roche, Mannheim, Germany, RRID: rid_000088). QuantiTect Primer Assays were used for iNOS (Mm_Nos1_2_SG), Il1b (Mm_Il1b_2_SG), Ym-1 (Mm_Chi3l3_1_SG), Arg1 (Mm_Arg1_1_SG), Olig2 (Mm_Olig2_1_SG), Snap25 (Mm_Snap25_2_DG) and Gapdh (Mm_Gapdh_3_SG), all QuantiTect Primer Assays from Qiagen.

### RNA sequencing

DNAase-treated bulk RNA samples isolated from PBS-perfused spinal cord tissues were quantified using NanoDrop ND-100 (ThermoFisher) and Bioanalyzer RNA 6000 Nano assay (Agilent). 500 ng of total RNA per sample of sufficient quality were then processed using the QuantSeq 3′ mRNA-Seq Library Prep Kit FWD (Lexogen) for library preparation. The libraries were then assessed for molarity and median library size using Bioanalyzer High Sensitivity DNA Analysis. The NGS was then be performed on an Illumina NextSeq550 Platform (Illumina), with a NextSeq 500/550 High Output Kit v2.5, 150 cycles, single read. Quality of the reads was evaluated using Fastqc v0.11.7 (available online at: http://www.bioinformatics.babraham.ac.uk/projects/fastqc/) while adapter and polyA trimming with length filtering (min = 30 bp) was performed using fastp [18]. Transcript abundance was quantified using Salmon v.1.1.0 [19] in quasi-mapping-based mode using the decoy aware Ensembl GRCm39 version 106 (mm39) and the recommended parameters for the lexogen protocol ‘–noLengthCorrection’ and ‘-l SF’. Raw counts were imported into RStudio (2023.03.0 Build 386) using tximport [20] and differentially expressed genes were identified using negative binomial models implemented in the DESeq2 package [21].

### Electrode implantation

Animals were anaesthetized with 0.8%–1.5% isoflurane and implanted with bipolar stimulating electrodes in the right Schaffer collateral/commissural pathway of the hippocampus, a recording electrode in the ipsilateral CA1 pyramidal layer and a ground wire affixed to the bone of the skull, as previously described by some of us [7, 22]. The final location of the recording electrode was confirmed by the presence of reliable monosynaptic fEPSPs evoked by paired pulses (40 ms interpulse interval) presented to Schaffer collaterals. After surgery, animals were returned to their home cages to recover at least 7 days before the beginning of the electrophysiology recordings.

### In vivo electrophysiology recordings

All the electrophysiology experiments were performed in adult (3–5 months old; 25–30 g) male mice under specific housing conditions (no females in the same building, sound- and vibration-proof). Animals were prepared according to procedures described elsewhere by some of us [22]. Animals were placed in separate, small (5 × 5 × 10 cm) plastic chambers located inside a larger Faraday box (30 × 30 × 20 cm). fEPSPs were recorded with the help of Grass P511 differential amplifiers (Grass Instruments, Warwick, RI, USA) through high-impedance probes (2 × 10^12^ Ω, 10 pF). Recording sessions were performed with a maximum of six animals at a time. All the in vivo recordings made in awake, non-anaesthetized animals were carried out in accordance with European Union (2010/63/EU) guidelines and Spanish (BOE 34/11370–421, 2013) regulations for the use of laboratory animals in chronic experiments. The experimental protocols were also approved by the local ethics committee of the Pablo de Olavide University (Seville, Spain).

### Input/output (I/O) curves

To investigate basal synaptic transmission properties, we measured input/output responses by stimulating the Schaffer collaterals with paired pulses (40 ms of inter-stimulus intervals) at increasing intensities (0.02–0.4 mA, in steps of 0.02 mA, 10 paired pulses for each intensity).

### Paired pulse facilitation

To evaluate pre-synaptic functions [23], we induced paired pulse facilitation (PPF), a form of short-term plasticity that measures the probability of neurotransmitter release from the presynaptic terminal. We checked the effects of paired pulses at different (10, 20, 40, 100, 200 and 500 ms) inter-stimulus intervals using intensities corresponding to 30–40% of the amount required to evoke a saturating response. In all cases, the pair of pulses of a given intensity was repeated at least five times with time intervals ≥ 30 s, to avoid as much as possible interference with slower short-term potentiation (augmentation) or depression processes [23].

### Long-term potentiation

To investigate post-synaptic functions, we induced long-term potentiation (LTP), a form of long-term plasticity that is thought to underlie the processes of learning and memory. To evoke LTP in behaving mice, we followed procedures described elsewhere [7, 22]. fEPSP baseline values were collected 15 min before LTP induction using paired (40 ms interstimulus intervals) 100 µs, square, and biphasic pulses. Pulse intensity was set at 35% of the amount necessary to evoke maximum fEPSP response (0.15–0.25 mA), which is below the threshold for evoking a large population spike. An additional criterion for selecting stimulus intensity was that the second stimulus should evoke a larger (> 20%) fEPSP than the first. For LTP induction, animals were presented with an HFS session consisting of five 200 Hz, 100 ms trains of pulses at a rate of 1 min. Thus, a total of 600 pulses were presented during an HFS session. To avoid evoking population spikes and/or the appearance of local seizures, the stimulus intensity during HFS was set at the same as that used for generating baseline recordings. Animals that presented discharges or motor seizures after the HFS protocol (as checked by online electroencephalographic (EEG) recordings and visual observation of the stimulated animal) were excluded from the study. The HFS session was repeated for the first 2 consecutive days. After the HFS sessions, the same paired-pulse stimuli (40 ms inter-stimulus interval) were presented every 20 s for 60 min and for 30 min the 4 following days. For the analysis of the data, we measured the amplitude of the first fEPSP recorded throughout the different sessions.

### Electrophysiology data analysis and representation

fEPSPs and 1 V rectangular pulses corresponding to lever presses, paired-pulse presentations and HFS were stored digitally on a computer through an analog/digital converter (CED 1401 Plus; Cambridge Electronic Design, Cambridge, England). Data were analyzed offline for quantification of animal performance in instrumental learning and fEPSPs with the Spike 2 (Cambridge Electronics Design, RRID: rid_000090) program and the video-capture system. Depending on the experiment, 5–15 successive fEPSPs were averaged, and the mean value of the amplitude during the rise-time period (i.e., the period between the initial 10% and the final 10% of the fEPSP) was determined. Computed results were processed for statistical analysis.

### Statistics

Statistical analyses were performed using SigmaPlot 11.0. Data are presented as mean ± SEM. P values are from two tailed Student’s t-tests (for normally distributed data) or Mann–Whitney *U* tests (for non-normally distributed data) or one-sample t-test. Normality of data was checked using the Kolmogorov–Smirnov or Shapiro–Wilk test and equality of variance was confirmed using the F-test. For multiple comparisons, *p* values are corrected using a procedure equivalent to the Holm–Bonferroni method (for N comparisons, the most significant *p* value is multiplied by N, the 2nd most significant by N-1, the 3rd most significant by N-2, etc.). The electrophysiology data were analyzed with 2-tailed Student’s t test or the 1-way or 2-way analysis of variance (ANOVA) using days as the repeated measurement followed by a contrast analysis (post hoc) for further analysis of significant differences. Values of *p* ≤ 0.05 were considered statistically significant.

## Results

### Global depletion of IKKβ from CNS and peripheral myeloid cells accelerates the onset and supresses the chronic phase of EAE

**Fig. 1:**
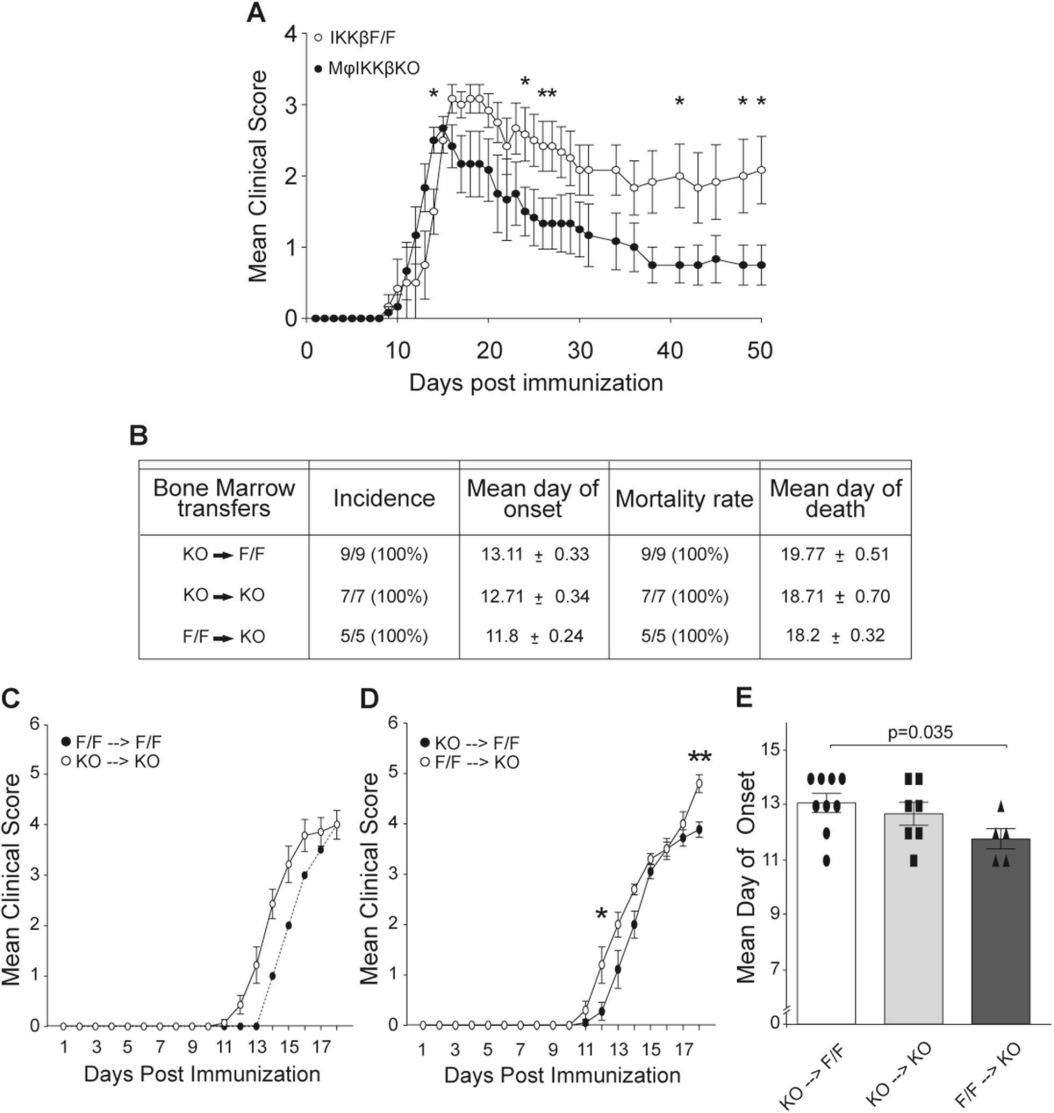
Global depletion of myeloid IKKβ results in slight earlier onset and significantly supressed chronic EAE. **A** Representation of the mean EAE clinical score for IKKβF/F and MφIKKβΚΟ female mice over 50 days post immunization with the peptide MOG_35-55_. **B** Table showing experimental parameters, such as incidence, mean day of onset, mortality rate and mean day of death, of the three different EAE experiments that took place in bone marrow chimeric groups that were generated from IKKβF/F (F/F) and mφIKKβΚΟ (KO) female mice. Specifically, MOG_35-55_-EAE was induced in two experimental groups of chimeric mice; (1) KO- > F/F with IKKβ depleted peripheral myeloid cells and IKKβF/F CNS macrophages, (2) F/F- > KO with IKKβF/F peripheral myeloid cells and IKKβ depleted CNS macrophages and in two controls; (1) KO- > KO with IKKβF/F depleted CNS macrophages and peripheral myeloid cells, and (2) F/F- > F/F with IKKβF/F CNS macrophages and peripheral myeloid cells. The F/F- > F/F group is not included in the table because only one mouse survived in this group and could not be statistically compared with the others. **C**, **D** Representation of the mean EAE clinical score for the chimeric group of mice F/F- > F/F, KO- > KO, KO- > F/F and F/F- > KO over 18 days post immunization with the peptide MOG_35-55_. **E** Comparison of the mean day of EAE onset between the chimeric group of mice KO- > F/F, KO- > KO and F/F- > KO. Numbers of mice are annotated as scatter dots on the bars. All mice were adult females 2–4 months old

We investigated the role of myeloid IKKβ kinase to the pathogenesis of EAE. We started by testing the effect of knocking out IKKβ from both CNS and peripheral myeloid cells, using constitutive MφIKKβKO mice. We crossed IKKβF/F [8, 9] with transgenic mice expressing Cre recombinase constitutively under the control of CD11b promoter. Previous characterization of these mice showed that the IKKβ protein levels were depleted in both MφIKKβKO peripheral macrophages (by 44%) and MφIKKβKO CNS macrophages (by 37%) compared with controls [7]. Induction of EAE in IKKβF/F and MφIKKβKO female mice by immunization with the MOG_35-55_ peptide in complete Freund’s adjuvant (CFA), followed by injection of pertussis toxin, resulted in slightly earlier onset and significantly reduced clinical symptoms in the chronic phase of disease in MφIKKβKO compared with IKKβF/F controls (dpi 25–50) in two out of three independent experiments (Fig. 1 and Additional file 1: Fig. S1A and B) and showed a tendency in the third (Additional file 1: Fig. S1C).

We next examined whether differences in peripheral immune responses to antigen might underlie the effect of myeloid IKKβF/F deletion on the clinical score. We initially analyzed T cell responses to Ag priming by immunizing mice with MOG_35-55_ or ovalbumin protein (OVA). Nine days later we isolated splenocytes and lymph node cells (DLN) and re-stimulated them in vitro with their respective cognate antigen, MOG_35-55_ at 1, 10 and 50 μg/mL (Additional file 2: Fig. S2A) or OVA at 1, 10 and 100 μg/mL, respectively (Additional file 2: Fig. S2B). Cells derived from MφIKKβKO, and control mice showed equal responses to MOG_35-55_ peptide and OVA at all concentrations tested (Additional file 2: Fig. S2A & B). We next measured cytokine secretion by FACS analysis in splenocytes and DLN of control and MφIKKβKO mice with MOG_35-55_ induced EAE, at a chronic phase of the disease (dpi 23). No differences in the production of IL-17, IFNγ, IL-10, IL-4, TNF or in Tregs between the two groups were detected (Additional file 2: Fig. S2C, D).

These results show that global depletion of IKKβ from peripheral and CNS myeloid cells has dual effects in EAE, on the one hand slightly accelerating the onset of the disease, while on the other markedly protecting in the chronic phase. EAE clinical symptoms were not associated with alterations in antigen-mediated T cell priming.

### Global depletion of IKKβ from CNS and peripheral myeloid cells resulted in earlier CNS tissue recovery in chronic EAE

**Fig. 2:**
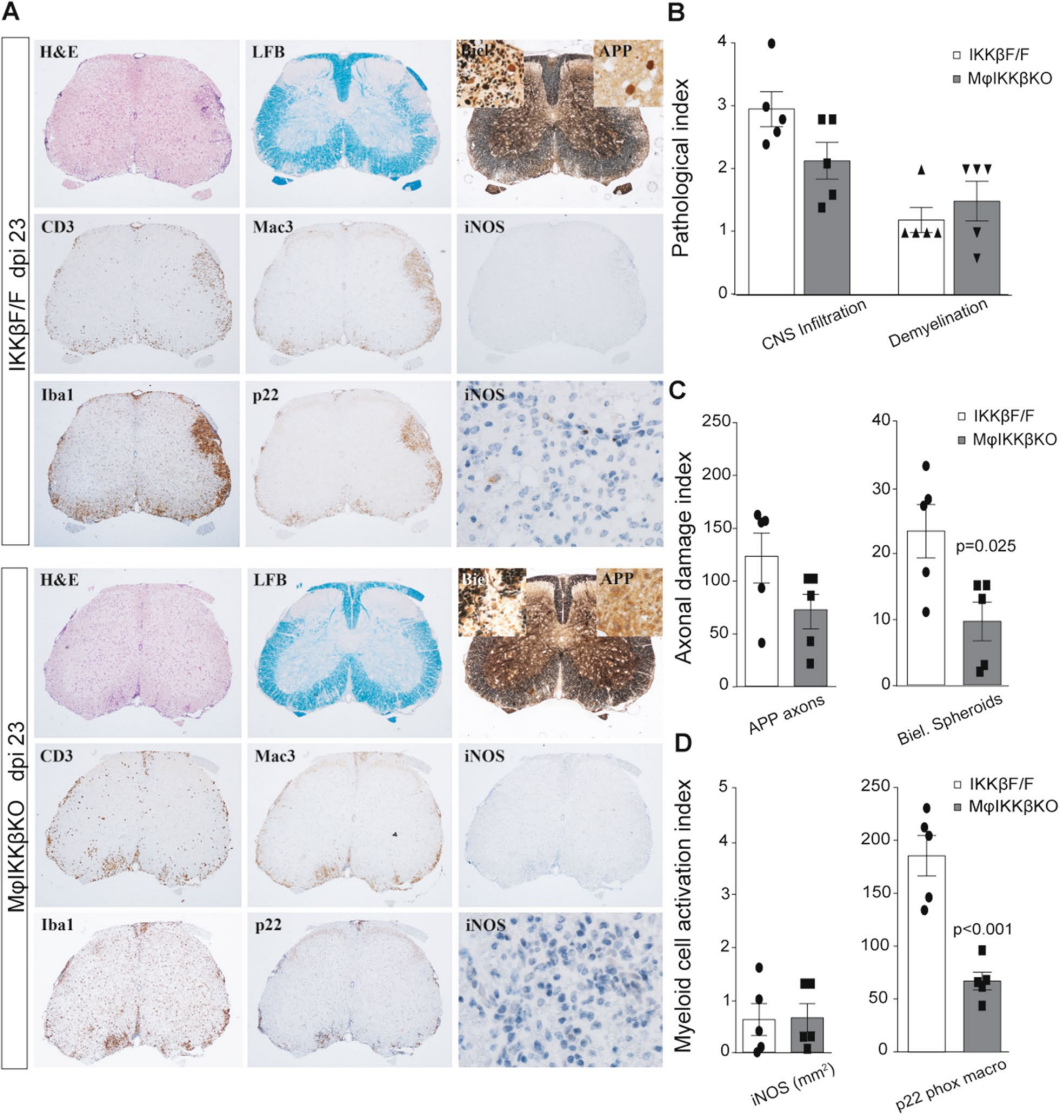
Global depletion of myeloid IKKβ results in reduced axonal damage and oxidative stress in EAE. **A** Specimen images of paraffin-embedded coronal spinal cord sections from IKKβF/F (upper panel) and ΜφΙΚΚβKO (lower panel) mice with MOG_35-55_-induced EAE at 23 dpi (chronic phase). Serial sections were stained for markers of inflammation and demyelination, such as hematoxylin and eosin (H&E) and Luxol fast blue (LFB), respectively. Representative images from immunohistochemistry for markers of axonal damage (APP and Biel. Spheroids), immune cell infiltration (CD3 and Mac3), myeloid cell activation (iNOS) and macrophagic oxidative stress (p22 phox) are also shown. **B** Semi-quantitative scoring of immune cell infiltration (increased intensity of H/E staining) and demyelination (loss of LFB staining) of the same groups of mice shown in **A**. **C** Quantification index of axonal damage measured as levels of axonal APP and number of Biel. Spheroids of the same groups of mice shown in **A**. **D** Quantification index of myeloid cell activation measured as levels of iNOS and number of macrophagic p22 phox of the same groups of mice shown in A. Numbers of mice are annotated as scatter dots on the bars. All mice were adult females 2–4 months old

We next investigated whether pathological features of EAE, such as neuroinflammation, demyelination and/or axonal integrity, were affected by constitutively knocking out IKKβ kinase from CNS and peripheral myeloid cells. Staining of spinal cord paraffin sections from control and mφIKKβKO mice at dpi 23 for markers of inflammation and demyelination, such as hematoxylin and eosin (H&E) and Luxol fast blue (LFB), respectively, showed that neither of these pathological indices were different among the two groups (Fig. 2A, B). Further immunohistochemistry for markers of axonal damage (APP and Biel. spheroids), immune cell infiltration (CD3 and Mac3), myeloid cell activation (iNOS and Iba1) and macrophagic oxidative stress (p22 phox) showed that the levels of Biel. spheroids and the number of p22 phox-positive macrophages were significantly lower in MφIKKβKO mice compared with controls, while the other measurements were equal between the two groups (Fig. 2A, C, D). Thus, the EAE clinical amelioration shown in MφIKKβKO mice compared with controls in the chronic phase of the disease was accompanied by less axonal damage coupled with reduced oxidative stress activity in the MφIKKβKO macrophages.

RT-PCR on bulk RNA samples isolated from spinal cords of control and MφIKKβKO mice at dpi 23 further showed that neither pro- or anti-inflammatory genes, such as iNOS or IL-1β and Arg-1 or Ym1 respectively, were differentially regulated between the two groups (Additional file 3: Fig. S3). However, genes involved in myelin and neuronal repair, such as Olig2 and Snap25, were significantly upregulated in the MφIKKβKO mice with EAE relative to naïve, and slightly more than in IKKβF/F controls (Additional file 3: Fig. S3). These data further suggest that depleting IKKβ globally from both CNS and peripheral myeloid cells renders macrophages less oxidative and protects mice in chronic EAE by reducing axonal damage and promoting CNS tissue recovery.

### Inducible deletion of IKKβ from CNS resident but not peripheral macrophages results in earlier onset and exacerbated chronic EAE

We next investigated the specific roles of CNS versus peripheral myeloid cell IKKβ in EAE. We first used a bone marrow chimera approach, which has frequently been used to distinguish the effects of brain macrophages from those of bone marrow-derived immune cells, due to the high radio-resistance of the former [24, 25]. Sub-lethally irradiated IKKβF/F or MφIKKβKO female recipient mice (doses in materials and methods) were transplanted with bone marrow cells isolated from either MφIKKβKO or IKKβF/F healthy donor mice. MOG_35-55_—EAE was induced in two experimental and two control groups of chimeric mice as follows: experimental groups: (1) MφIKKβKO bone marrow cells transplanted into IKKβF/F recipient mice (KO- > F/F); (2) IKKβF/F bone marrow cells transplanted into MφIKKβKO recipient mice (F/F- > KO). Control groups: (1) KO- > KO, (2) F/F- > F/F. F/F- > KO mice, which lacked IKKβ only in CNS myeloid cells, developed EAE earlier than KO- > F/F mice, which lacked IKKβ only in peripheral myeloid cells (Fig. 1B–E). This suggests that the IKKβ in CNS macrophages protects mice against the clinical onset of EAE. The differential effects of depletion of IKKβ from CNS or peripheral myeloid cells in the chronic phase of EAE could not be addressed by this approach as both chimeric groups developed severe clinical symptoms after the peak of disease and experiments were stopped early (Fig. 1B). Similarly, due to the high mortality rate, only one mouse survived in the F/F- > F/F group and this could not be statistically compared with the other groups (Fig. 1C).

Studies have shown that CNS macrophages are not completely resistant to whole body gamma irradiations [26]. Furthermore, although they are less susceptible to irradiation-induced cell death than peripheral macrophages, some of them, particularly the CNS parenchymal microglia, become activated early after X-irradiation showing decreased cell numbers and changes in morphology [27]. Thus, we next used a genetic approach to confirm and extend the results obtained using bone marrow chimeras. Specifically, we crossed IKKβF/F mice with tamoxifen-inducible Cx3cr1-Cre^ER+/−^ mice to generate double Cx3cr1-Cre^ER+/−^IKKβF/F (or MgIKKβKO) knockout mice. To differentiate the role of IKKβ of CNS macrophages versus other tissue CX3CR1^+^ myeloid cells we adopted an established tamoxifen induction protocol that exploits the different origins and turnover rates of the two groups [28, 29]. We administered tamoxifen for 2 or 4 consecutive days (as indicated in each experiment in figure legends) followed by a 5-day waiting period, during which IKKβ is depleted in both CNS and peripheral macrophages, or a 28-day waiting period, during which the fast-renewing macrophages are replenished by wild-type bone marrow progenitors while the self-maintaining CNS parenchymal macrophages population is not replenished and maintain IKKβ ablation. The gene targeting specificity in this system is described in materials and methods and in Additional file 4: Fig. S4 and Additional file 5: Fig. S5.

**Fig. 3:**
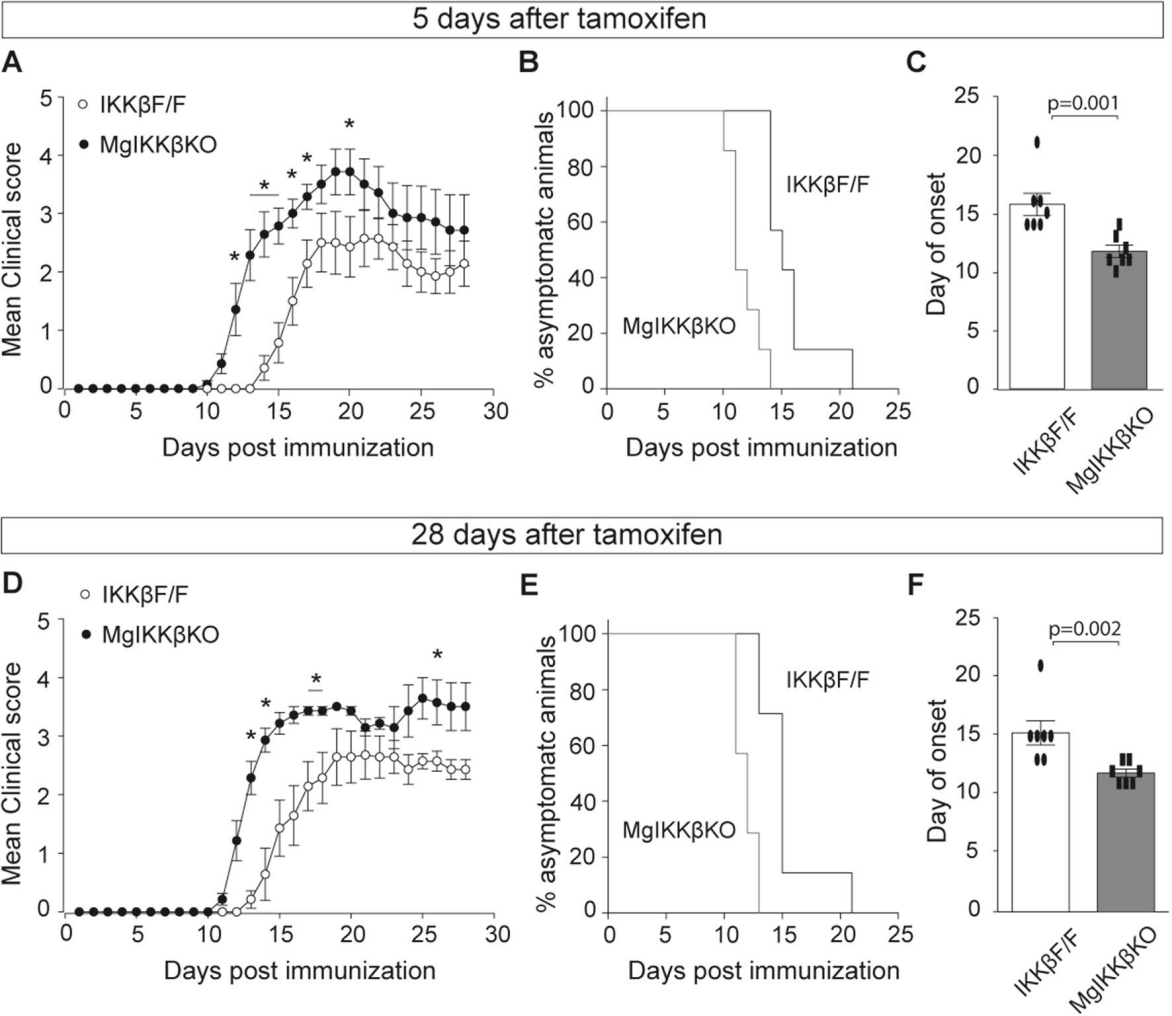
Knocking out IKKβ selectively from CNS macrophages results in earlier onset and exacerbated chronic EAE. **A**–**C** Comparison of EAE between IKKβF/F and ΜgΙΚΚβΚΟ mice that was induced at 5 days after tamoxifen (4 doses), when IKKβ was depleted from both CNS and peripheral myeloid cells. **A** Representation of the mean EAE clinical score over 30 days post-immunization with the peptide MOG_35-55_. **B** Mantel Cox graph showing the proportion (%) of asymptomatic IKKβF/F and ΜgΙΚΚβΚΟ animals over 25 days post immunization with the peptide MOG_35-55_. **C** Comparison of the mean day of EAE onset between the IKKβF/F and ΜgΙΚΚβΚΟ mice. **D**–**F** Comparison of EAE between IKKβF/F and ΜgΙΚΚβΚΟ mice that was induced at 28 days after tamoxifen (2 doses), when IKKβ was depleted only from CNS myeloid cells. **A** Representation of the mean EAE clinical score over 30 days post-immunization with the peptide MOG_35-55_. **B** Mantel Cox graph showing the proportion (%) of asymptomatic IKKβF/F and ΜgΙΚΚβΚΟ animals over 25 days post immunization with the peptide MOG_35-55_. **C** Comparison of the mean day of EAE onset between the IKKβF/F and ΜgΙΚΚβΚΟ mice. Numbers of mice are annotated as scatter dots on the bars. All mice were adult females 2–4 months old

We used the tamoxifen inducible MgIKKβKO system to investigate the effects of knocking out IKKβ selectively from CNS macrophages upon the pathology of EAE. The disease was induced in female MgIKKβKO and IKKβF/F control mice at 5 and 28 days after tamoxifen to reveal the effect of knocking out IKKβ to both CNS and peripheral myeloid cells and to only CNS macrophages, respectively. MgIKKβKO mice in which EAE was induced 5 days after tamoxifen developed EAE with a significant earlier clinical onset compared with IKKβF/F mice, although the difference among the two groups was diminished at the chronic phase of the disease, possibly due to the progressively diminished IKKβ depletion in peripheral myeloid cells as disease progressed (Fig. 3A–C). MgIKKβKO mice in which EAE was induced at 28 days after tamoxifen developed EAE with a significant earlier clinical onset compared to IKKβFF mice as before, however the difference among the two groups in this case remained significant also during the chronic phase of the disease, with the MgIKKβKO mice showing worse clinical symptoms compared to IKKβF/F (Fig. 3D–F). Control EAE experiments using IKKβF/F and MgIKKβKO mice that had not been treated with tamoxifen showed no differences in the clinical score between these two groups (Additional file 5: Fig. S5C), suggesting that the tamoxifen treatment by itself does not alter the disease outcome. Furthermore, EAE was not different between the heterozygous CX3CR1-Cre^+/−^ and the IKKβF/F mice, but both were statistically different compared to the MgIKKβΚΟ mice (Additional file 5: Fig. S5D), suggesting that heterozygosity of the CX3CR1 gene in the MgIKKβΚΟ mice is not accountable for the earlier onset of EAE seen in these mice, an effect that resulted only from depletion of myeloid IKKβ.

Overall, the data so far show that knocking out IKKβ from myeloid cells affect EAE in opposing ways depending on the cell type and the stage of the disease. Constitutive knocking out of IKKβ in all myeloid cells leads to slightly earlier onset and to a strong and significant amelioration of the clinical pathology during the chronic phase of the disease. On the other hand, inducible depletion of IKKβ selectively in CNS and not in peripheral macrophages resulted in exacerbated clinical pathology at all stages of EAE, including a significantly earlier disease onset. These observations suggest that knocking out IKKβ from peripheral macrophages is protective in chronic EAE, in contrast to knocking it out from CNS macrophages, which has deleterious effects.

### Inducible deletion of IKKβ selectively from CNS resident macrophages resulted in earlier demyelination and immune cell infiltration in EAE but had no effect in cuprizone-induced demyelination

**Fig. 4:**
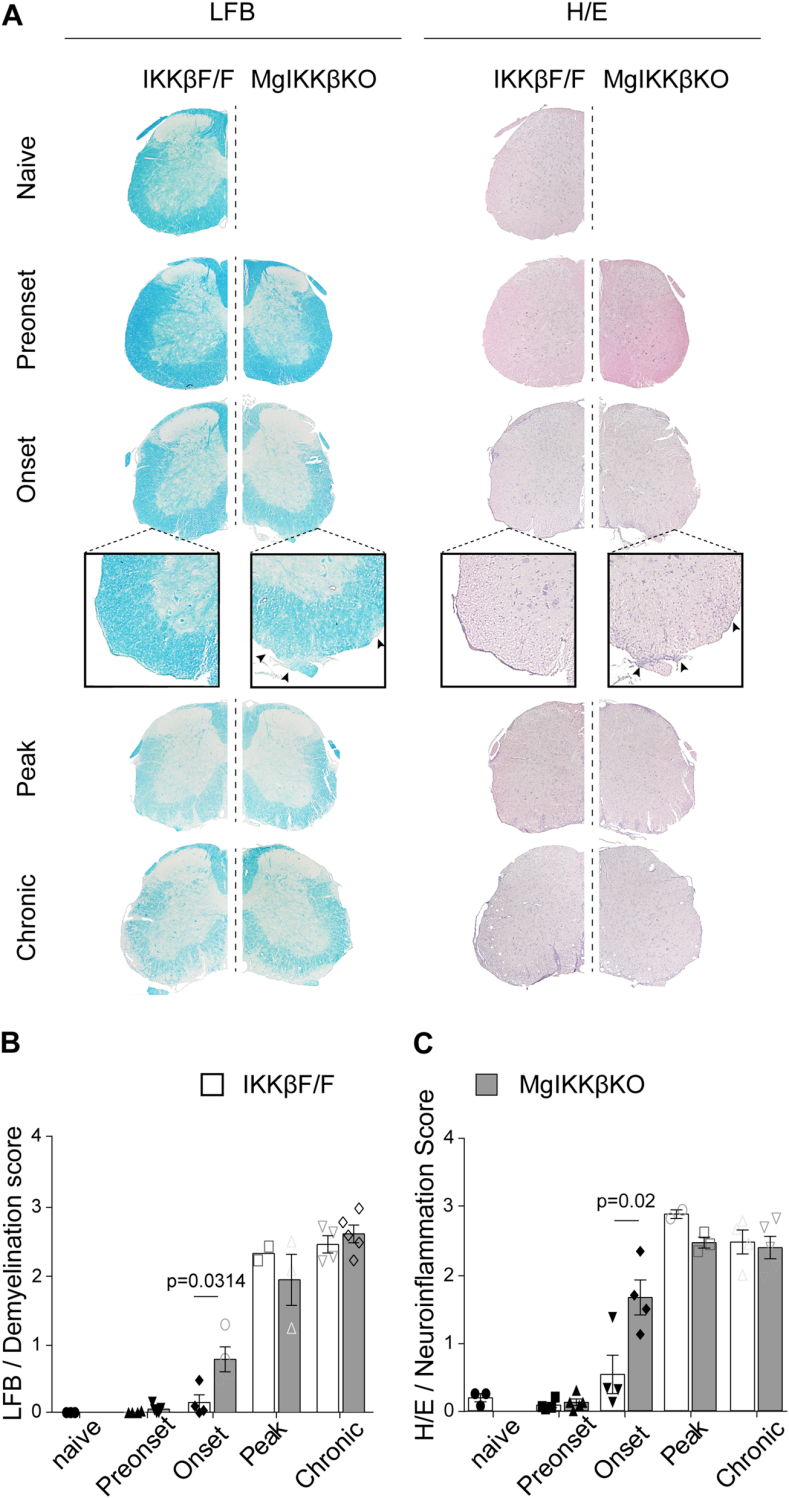
Knocking out IKKβ selectively from CNS macrophages results in early demyelination and neuroinflammation in EAE. **A** Specimen images of paraffin-embedded coronal spinal cord sections from IKKβF/F and ΜgΙΚΚβKO mice stained with LFB (left column) and H/E (right column) showing the evolution of demyelination and immune cell infiltration, respectively, during MOG_35-55_-induced EAE. Specifically, in the naïve spinal cord, at a pre-onset stage (dpi 8), at the onset (of the ΜgΙΚΚβKO mice), at the peak and at the chronic phase of the disease. **B** Semi-quantification of the mean demyelination level in spinal cord of IKKβF/F and ΜgΙΚΚβKO mice shown in **A**, left column. **C** Semi-quantification of the mean immune cell infiltration level in spinal cord of IKKβF/F and ΜgΙΚΚβKO mice shown in **A**, right column. Numbers of mice are annotated as scatter dots on the bars. All mice were adult females 2–4 months old

We then tested whether the exacerbated clinical EAE disease in MgIKKβKO mice was associated with enhanced neuroinflammation and/or demyelination. IKKβF/F and MgIKKβKO mice, at 28 days after tamoxifen so that IKKβ was depleted only from CNS macrophages, were immunized for EAE or left non-immunized (naïve) and killed at different time-points of the disease, at dpi8 (pre-onset), onset (of the MgIKKβKO mice), peak and chronic phases. Mice were perfusion-fixed and spinal cords were isolated and processed for standard histology in paraffin sections. Demyelination and immune cell infiltration in lumbar spinal cords were examined by LFB and H/E staining (Fig. 4A), respectively. Both pathological indices were higher in the MgIKKβKO mice compared with controls (IKKβF/F) at the onset of the disease in the KO group, but not at the other time-points tested (Fig. 4B, C). These data show that knocking out IKKβ in CNS macrophages results in earlier onset of EAE, that is accompanied by more neuroinflammation and demyelination.

We then investigated whether knocking out IKKβ selectively from CNS macrophages might affect pathology of another MS model, cuprizone-induced demyelination. Twenty-eight days after tamoxifen, male MgIKKβKO and IKKβF/F mice were fed with cuprizone (0.2% w/w) for a total period of 6 weeks and thereafter were put on a normal chow for another 2 weeks, for remyelination to occur. We perfusion fixed them at different time points selected based on pathological hallmarks characterized in the white matter of the corpus callosum [14], i.e., at 3 and 5 (peak of demyelination) weeks of cuprizone feeding, and remyelination at 2 weeks after removal of cuprizone from the diet (6 + 2 weeks). Demyelination in the midline corpus callosum was measured by LFB staining of paraffin sections from IKKβF/F and MgIKKβKO mice at the different experimental time points (Additional file 6: Fig. S6A). LFB staining was reduced and less evenly distributed in the corpus callosum of both groups at week 5, consistent with demyelination (Additional file 6: Fig. S6A, D). Microgliosis and astrogliosis were also measured in paraffin sections in midline corpus callosum of IKKβF/F and MgIKKβKO mice by quantifying the levels of Iba-1 and GFAP immunoreactivity, respectively (Additional file 6: Fig. S6B, C). Both immunoreactivities were altered similarly in the two groups at all time points (Additional file 6: Fig. S6E, F). Thus, IKKβ depletion from CNS macrophages did not induce detectable changes in white matter pathology or recovery in the cuprizone model.

Overall, these data show that IKKβ in CNS resident macrophages delays the clinical onset of EAE and associated pathology, neuroinflammation and demyelination, but does not detectably affect toxin-induced demyelination and remyelination. Together the results suggest that CNS macrophagic NF-κB activity might protect against EAE by inhibiting immune cell infiltration into the CNS microenvironment.

### CNS resident macrophage rather than peripheral T cell effector functions accelerate EAE onset after knocking out IKKβ

We next investigated whether knocking out IKKβ selectively from CNS resident macrophages might affect T cell activation and effector function, which is critical for initiating EAE. IKKβF/F and MgIKKβKO mice, at 28 days after tamoxifen, were subjected to EAE and killed at dpi12, which corresponded to the clinical onset in the MgIKKβKO mice (Additional file 7: Fig. S7A). FACS was then performed on cells isolated from CNS (spinal cord) and peripheral (spleen and lymph node) tissues that were co-labeled for the T cell marker CD4 together with other key inflammatory markers, such as IFNγ, IL-17, GM-CSF, TNF and CD44. The analysis showed that the number of infiltrating CD4 + T cells in the CNS was higher in the MgIKKβKO mice compared with controls, as expected from the earlier clinical onset of these mice (Additional file 7: Fig. S7B). However, although higher in total numbers, the CD4 + T cells in the CNS of the MgIKKβKO mice did not show altered expansion of effector T cell subpopulations as judged by the percentage of CD4 + T cells that were also positive for IFNγ and IL-17, which was similar between the MgIKKβKO and IKKβF/F mice (Additional file 7: Fig. S7B). Similarly, in the periphery, both in spleens and lymph nodes, the proportions of CD4 + T cells that were also positive for IFNγ, IL-17, GM-CSF, TNF and CD44 was similar between the two groups (Additional file 7: Fig. S7C, D). These data show that the observed exacerbation of clinical disease in MgIKKβKO mice is not due to alterations in activation and differentiation of effector T cells.

To examine whether CNS intrinsic mechanisms may rather be affected by knocking out IKKβ from CNS macrophages in EAE we focused on microglia, a unique ramified type of CNS macrophages that reside into the tissue parenchyma and respond rapidly to alterations in their microenvironment upon pathology, by changing morphology, kinetics and/or transcriptomic phenotype [30]. We investigated whether microglia morphology may be different in the MgIKKβKO spinal cord tissues compared with controls in the early stages of EAE, that could imply differences in their activation status. For this reason, we performed fluorescent immunohistochemistry on cryostat spinal cord sections from perfusion-fixed mice and acquired high-resolution confocal images of microglia labeled for Iba1. We then performed morphological analysis on a single-cell level, using a method based on MicroApp, a semi-automated method to analyze microglia morphology on a cell basis that we have previously described [15]. In brief, Iba-1 positive microglial cells were isolated manually from the confocal stacks using Image J, computationally traced by Imaris and analyzed for a series of parameters, such as the total process length, volume, diameter, etc. In this study, we used hypertrophic index, a ratio of total volume / total process length, as marker of microglial activation in EAE compared with naïve.

**Fig. 5:**
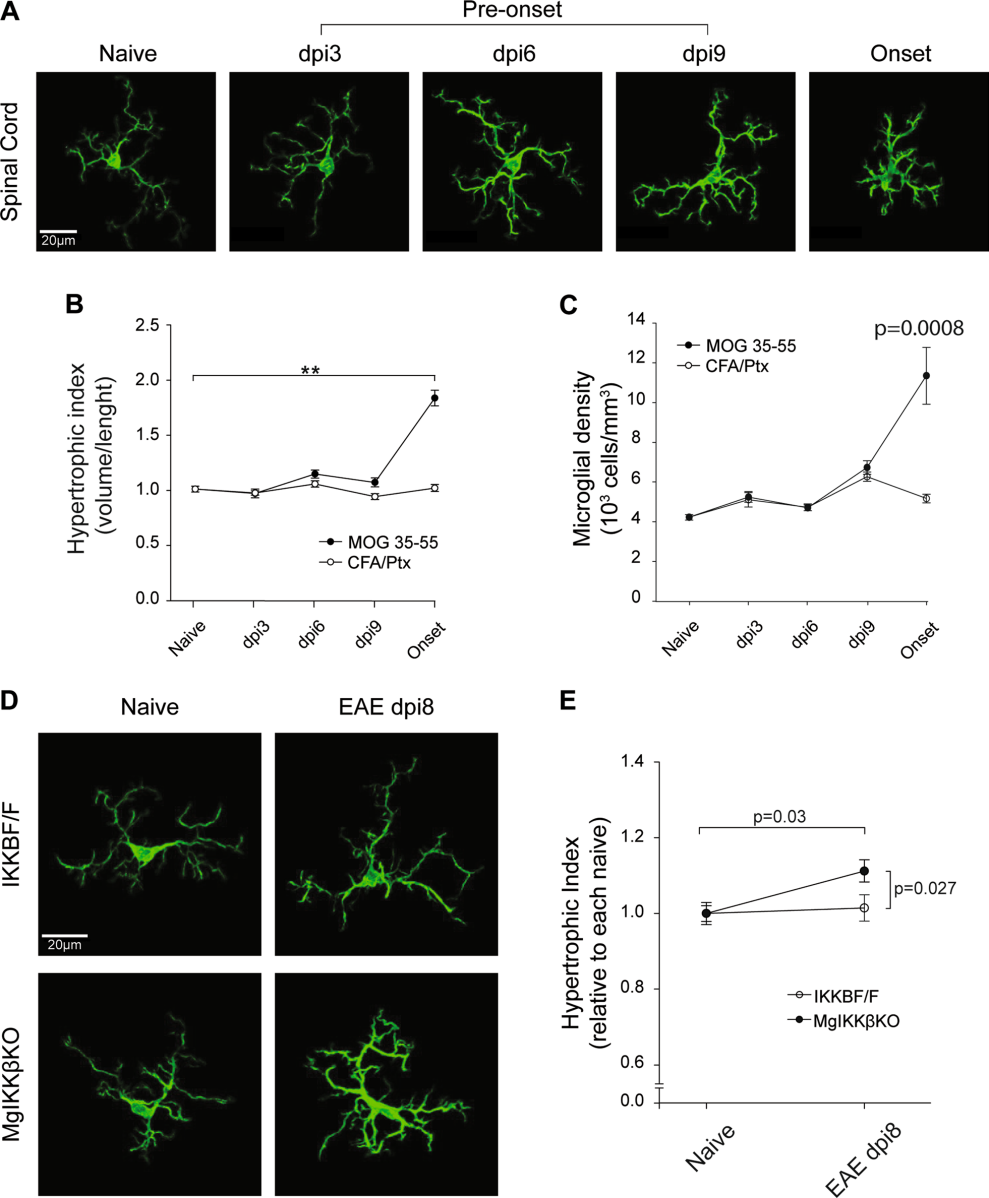
Spinal cord parenchymal microglia change morphology at the pre-onset stage of EAE in ΜgΙΚΚβKO mice. **A** Representative confocal images of individual microglia from cryostat slices immunolabeled for Iba-1 showing the spatiotemporal evolution of microglial morphology at the spinal cord of female C57BL/6 mice during MOG_35-55_-induced EAE. Specifically in the naïve spinal cord, at a pre-onset stage (dpi 3, 6 and 9) and at the onset of the disease. **B** Quantification of the microglial relative hypertrophic index (volume/length) in the spinal cord of C57BL/6 mice mice with MOG_35-55_-induced EAE or in controls that had been injected with the CFA adjuvant only and the Pertussis toxin (CFA/Ptx). **C** Quantification of microglial density in the spinal cord of C57BL/6 mice mice with MOG_35-55_-induced EAE or in controls that had been injected with CFA adjuvant only and Pertussis toxin (CFA/Ptx). **D** Representative confocal images of individual microglia from cryostat spinal cord slices from IKKβF/F and ΜgΙΚΚβKO mice that were either naïve or at the dpi 8 pre-onset stage of MOG_35-55_-induced EAE. **E** Quantification of the microglial relative hypertrophic index in the spinal cord of naïve and EAE dpi8 IKKβF/F and ΜgΙΚΚβKO mice. All mice were adult females 2–4 months old

Given the lack of previous published assessment of microglial activation at pre-clinical stages of EAE, we first performed longitudinal measurements of microglial activation in the grey matter of spinal cord tissues of control C57BL/6 female mice at different time points of EAE, including three pre-onset stages (dpi3, 6 and 9) and the onset of the disease (Fig. 5A). The analysis showed that the hypertrophic index of microglia was significantly increased only at the onset of MOG-induced EAE in these mice and not in other pre-clinical time points of the disease tested, and not at any time point in control mice, which were mice immunized with CFA alone and injected with Pertussis toxin (CFA/Ptx group) (Fig. 5B). We also measured microglia density and found that it was statistically increased to the spinal cord of C57BL/6 mice at the onset of EAE (Fig. 5C). We next performed similar analysis of microglia morphological alterations in MgIKKβKO and IKKβF/F mice with MOG_35-55_ induced EAE, at 28 days after tamoxifen. The hypertrophic index was measured in naïve mice of each group and at a pre-onset stage (dpi8) of EAE (Fig. 5D) and found to be significantly higher in the MgIKKβKO mice compared with controls in this timepoint (Fig. 5E). These data show that knocking out IKKβ selectively from CNS resident myeloid cells renders microglia prone to early morphological re-arrangements in response to EAE, even at the pre-onset stages of the disease, which might be related to the earlier clinical onset of the disease in these mice and needs further investigation.

Collectively, these data show that the exacerbation of clinical EAE in MgIKKβKO mice is not associated with alterations in peripheral effector T cell maturation or expansion but rather due to CNS resident mechanisms, as shown by differences in parenchymal microglial morphology at the pre-clinical stages of the disease, that imply differences in the activation status of these cells. However, the functional consequences of the observed differences in microglia morphology in the absence of NF-κB activation, and how they affect an autoimmune attack of CNS tissues, remains to be determined.

### RNA sequencing analysis of spinal cord from MgIKKβKO and IKKβF/F mice at pre-onset EAE

**Fig. 6:**
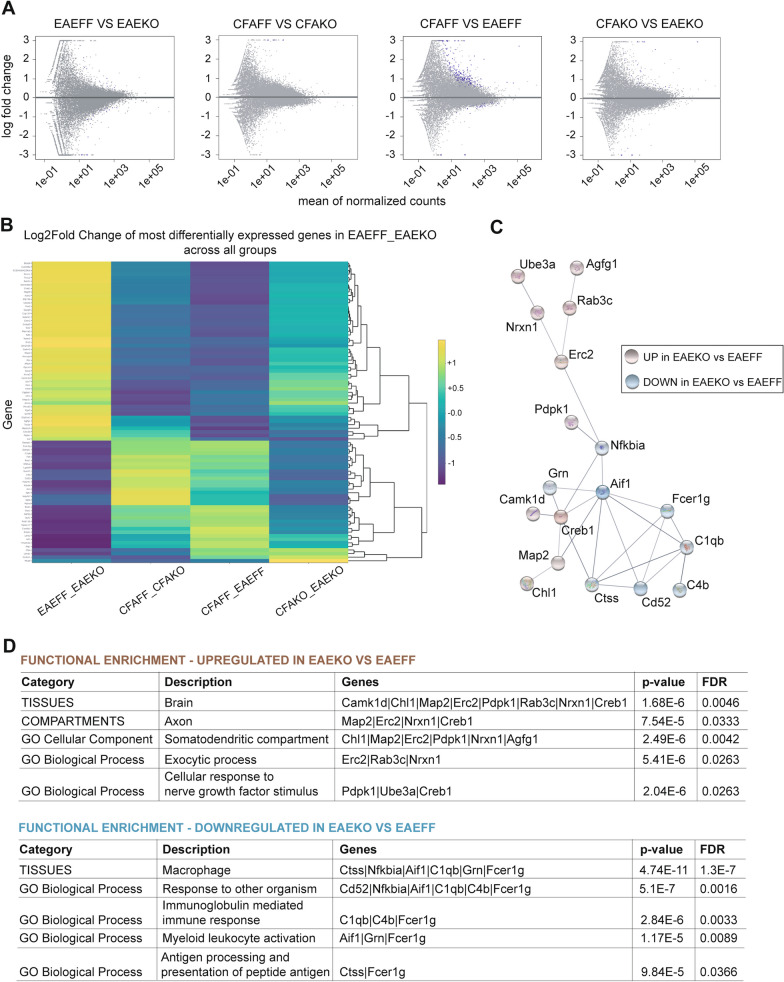
Depletion of IKKβ from CNS macrophages results in macrophagic and neuronal gene alterations in EAE. Summary of the RNA-seq results. **A** Volcano plot representation of the DEGs per comparison. Blue dots represent significant (Padj < 0.05) log2FoldChanges. The x-axis shows the normalized counts per gene and the y-axis shows the Log2FoldChange. **B** Heatmap of the most significant DEGs in the EAEFF_EAEKO group across all other groups. Values have been scaled (R package heatmaply [31]) for a better visualization. **C** PPI network of the most significant DEGs in the EAEFF_EAEKO group. Blue color nodes represent down-regulation of the gene while red color nodes represent upregulation

To further investigate how knocking out IKKβ from CNS macrophages affects the onset of EAE at a molecular level, we performed RNA sequencing on RNA samples isolated from spinal cord of female MgIKKβKO and IKKβF/F mice with MOG_35-55_ induced EAE at 28 days after tamoxifen (EAE_FF vs EAE_KO group). We performed the analysis on total RNAs isolated from spinal cord tissues at dpi8 of EAE, which is a pre-onset stage of the disease that, as we found above (Fig. 5D, E) and further measured for this experiment (Additional file 8: Fig. S8A & B), microglia already show increased hypertrophic index in response to MOG in the MgIKKβKO mice and not in controls IKKβF/F. This early microglial response cannot be a direct response to peripheral cell infiltration into the CNS parenchyma, as judged by the lack of immunofluorescent CD45^+^ immune cell infiltration into the spinal cord tissue of both MgIKKβKO and IKKβF/F mice (Additional file 8: Fig. S8C). Thus, we could make valid comparisons between the groups regarding the contribution of CNS intrinsic in the absence of peripheral immune cell involvement at the onset of EAE. However, we cannot rule out that there is already production and release of proinflammatory cytokines into the CNS parenchyma by meningeal infiltrates at this pre-onset stage of the disease. We also performed the same treatment and analysis to control groups of mice, MgIKKβKO and IKKβF/F, which were immunized only with the CFA adjuvant and injected with Pertussis toxin (CFA_KO vs CFA_IKKβF/F). The analysis showed that at dpi8, only few genes were statistically different between the compared groups (EAE_FF vs EAE_KO; CFA_FF vs CFA_KO; CFA_FF vs EAE_FF and CFA_KO vs EAE_KO) (Fig. 6A), which implies that the CNS tissue at this early pre-clinical phase of the disease is not dramatically altered at a transcriptomic level.

We then selected the most significantly differentially expressed genes (with Benjamini–Hochberg false discovery rate (Padj < 0.2) in the EAE_FF vs EAE_KO group and generated a heatmap of the log2fold change of these genes across all other compared groups to assess whether the differences were specific to MOG-induced EAE and not to CFA or Pertussis toxin treatment (Fig. 6B). The less conservative Padj cut-off (20% instead of 5%) was chosen in order to increase the power to detect biological differences and affected cellular mechanisms. This representation showed that the genes that are the most differentially expressed, either up- or down- regulated in EAE_KO vs EAE_FF, were different to those seen when compared groups that contained only CFA, suggesting that any transcriptomic differences seen in the EAE_FF vs EAE_KO group are solely due to the disease. Next, using the Cytoscape platform and the StringApp plugin, we constructed a protein–protein interaction (PPI) network by importing the aforementioned gene set and selecting the largest PPI network (with confidence score cutoff  ≤ 0.30) on which we performed a functional enrichment network analysis. Of note, we found that the upregulated genes in the EAE_KO vs EAE_FF group, such as *Nrxn1*, *Creb1*, *Map2*, *Ube3a*, *Erc2*, etc., were related to brain tissue and to neuronal dendrites and activities, neurotransmitter release and cellular responses to growth factors. On the other hand, the downregulated genes, such as Nfkbia, Aif1, Fcer1g, C4b, C1qb, etc., were related to macrophages, myeloid leukocyte activation, immune response, antigen processing and presentation of peptide antigen (Fig. 6C, D).

These data show that although the transcriptomic differences in the CNS of mice detected at the pre-onset stage of EAE is low, in the absence of IKKβ from CNS macrophages the gene networks that are mostly differentially regulated in response to MOG in the pre-clinical phase of the disease are related to both macrophagic and neuronal activities. Of note, the inflammatory macrophagic gene networks are reduced while those involved in neuronal functions related to neurotransmission are enhanced in the MgIKKβΚΟ mice at the pre-clinical phase of EAE which suggests that the homeostatic control of neuronal excitability by CNS macrophages in early EAE may be an important regulatory checkpoint for preserving the CNS tissue integrity and delaying the onset of the disease. The downregulation in the set of genes related to macrophagic functions at a pre-onset stage of EAE is somewhat expected in mice having the inflammatory action of the NF-κB transcription factor depleted in CNS macrophages, however is opposite to what expected from the worse EAE clinical disease of these mice compared with controls. The bulk RNA analysis on whole spinal cord tissues revealed changes in neuronal systems but did not allow prediction of CNS macrophages-specific mechanisms in the absence of CNS macrophagic IKKβ. Future single cell RNA approaches will be necessary to reveal whether depletion of myeloid NF-κB activity might affect the steady-state levels of homeostatic and/or disease-related genes in EAE.

### Global depletion of IKKβ from CNS and peripheral myeloid cells resulted in exacerbated neuronal excitability throughout EAE, starting from the pre-clinical phase of the disease

**Fig. 7:**
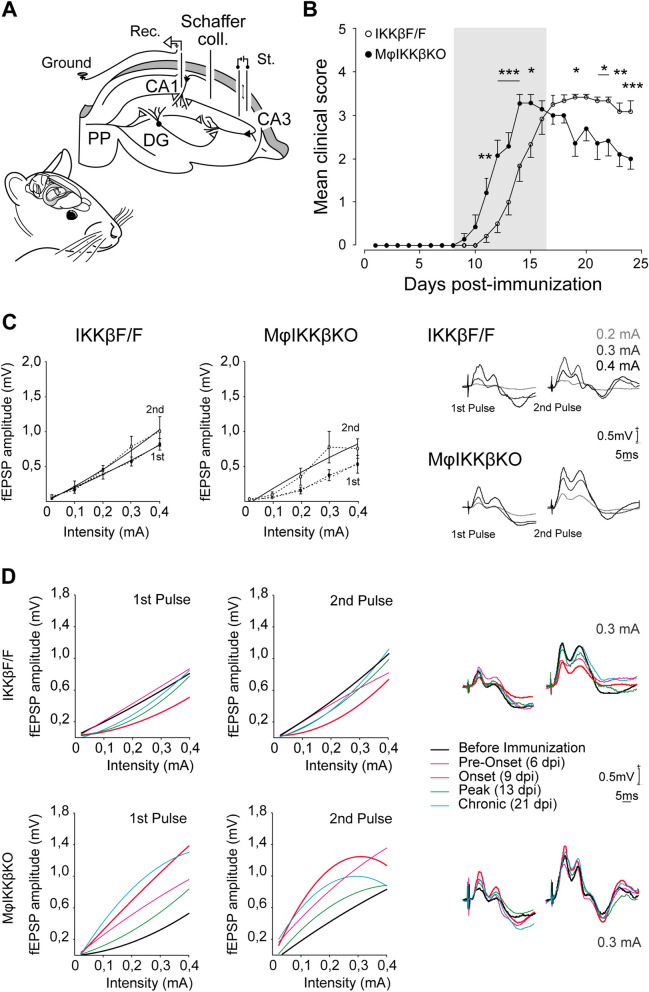
Global depletion of IKKβ from myeloid cells results in exacerbated neuronal excitability in EAE. **A** Bipolar stimulating electrodes (St.) were chronically implanted in the CA3 area of the right hippocampus to activate the Schaffer collateral/commissural pathway (Schaffer coll.). Recording electrodes (Rec.) were implanted in the ipsilateral CA1 area. **B** Representation of the mean EAE clinical score for IKKβF/F and MφIKKβΚΟ male mice with implanted electrodes, over 25 days post immunization with the peptide MOG_35-55_. **C** Regression lines illustrating input/output curves evoked at the CA3–CA1 synapse of IKKβF/F (*n =* 6) and MφIKKβKO (*n =* 5) mice. Paired pulses (40 ms of interstimulus interval) were delivered at increasing intensities in 20 μA steps. At the right are illustrated representative examples of fEPSP recordings (evoked at 0.2, 0.3 and 0.4 mA) of IKKβF/F and MφIKKβΚΟ mice. **D** Regression lines illustrating input/output curves evoked at the CA3–CA1 synapse of IKKβF/F (*n =* 6) and mφIKKbKO (*n =* 5) mice under naïve conditions (before immunization—black) and during MOG_35-55_-induced EAE, specifically at a pre-onset stage (dpi 6—pink), at the onset (dpi 9—orange), at the peak (dpi 13—green) and at a chronic (dpi 21—blue) phase of the disease. Representative examples of fEPSP recordings (0.3 mA) of IKKβF/F and MφIKKβΚΟ mice are also illustrated. All mice in this graph were adult males, 3–5 months old

To look further at the effect of myeloid cell NF-κB activity on neurons at a functional level during EAE, we performed in vivo electrophysiology experiments. We used the constitutive MφIKKβΚΟ mice that showed slightly earlier onset compared to IKKβF/F controls but also protection in chronic EAE (Fig. 1A), which was important for making direct correlations between neuronal alterations and disease progression and amelioration mechanisms. We particularly measured the functional properties of the hippocampal CA3–CA1 synapse in vivo, by stimulating the Schaffer collateral pathway in freely moving MφIKKβKO and IKKβF/F male mice, before and after EAE induction (dpi 25). To measure synaptic integrity in this pathway, electrophysiological recordings were taken from mice in which the stimulating and recording electrodes had been permanently implanted in the CA3 (Schaffer collaterals) and ipsilateral CA1 areas of the right hippocampus, respectively (Fig. 7A). Animals were allowed to recover at least 7 days after the implantation of the electrodes and before the electrophysiological recordings and the EAE induction. This experiment was performed in male mice due to the specific housing conditions of the laboratory that took place, which required no females in the same building for the purposes of behavioral studies (see Materials and methods). EAE induction in male MφIKKβKO mice resulted in suppressed chronic EAE, as expected from the same experiment in females, and further revealed a significant earlier onset compared with IKKβF/F controls (Fig. 7B), which was only a trend in females (Fig. 1A and Additional file 1: Fig. S1). This finding adds up to our previous data [7] showing a regulatory role of CNS macrophagic IKKβ in the onset of EAE and further reveal a sex factor to this effect.

We first measured synaptic transmission (input–output test) by recording changes in fEPSP amplitudes evoked in the pyramidal CA1 area by paired-pulse (40 ms of interpulse interval) stimulation of increasing intensity (0.02–0.4 mA, in 0.02 mA steps). We performed electrophysiological recordings one day before immunizations (before EAE), at 2 pre-clinical time points (dpi 6 and dpi 9), at the peak (dpi 13) and at the chronic phase (dpi 25) of EAE. Before EAE in IKKβF/F and MφIKKβKO mice, fEPSP amplitude evoked by the first and second pulses increased steadily (Fig. 7C). In MφIKKβKO mice fEPSP amplitude evoked by first pulse was similar to controls but the synaptic response from the second pulse tended to be higher at lower stimulation intensities (Fig. 7C). Although these differences were not statistically significant, these data are in accordance with our previous study [7] showing that MφIKKβKO mice have altered synaptic properties in the brain under physiological conditions. After EAE, in IKKβF/F mice fEPSP recordings evoked by first and second pulses tended to be equal or lower at all time points of EAE tested compared to the pre-immunization, time point (Fig. 7D, black line), with the recordings at EAE onset being the lowest (Fig. 7D, red line) and those at the chronic (dpi 13) and recovery (dpi 21) phases returning to pre-immunization levels. In contrast, in MφIKKβKO mice fEPSP recordings evoked by first and second pulses tended to be higher at all time points of EAE tested compared to pre-immunization (Fig. 7D, black line), including pre-onset (dpi 6, pink line), onset (dpi 9, red line), peak (dpi 12, green) and chronic (dpi 21, blue) phases (Fig. 7D).

We then evaluated presynaptic function at the hippocampal CA3–CA1 synapse using the paired-pulse facilitation (PPF) test, a form of short-term plasticity. Here, presynaptic neurons receive a pair of stimuli in rapid succession causing a transient accumulation of calcium in presynaptic nerve terminals. The increase in the ratio of the second/first fEPSP response (facilitation) at short (< 60 ms) intervals reflects mainly the increase in presynaptic calcium which in turn increases the probability of neurotransmitter release. We stimulated MφIKKβKO and IKKβF/F mice with paired pulses using a wide range of interstimulus intervals (20–500 ms) before immunizations and at two time-points after EAE induction, pre-onset (dpi6) and chronic phase (dpi 12). Both groups presented similar increase in response to the second pulse at the short time intervals (20 ms and 40 ms) at all time points tested (Additional file 9: Fig. S9A and B), demonstrating that in EAE myeloid IKKβ does not affect short-term plasticity.

These data suggest that in EAE myeloid cell NF-κB activity regulates synaptic transmission by suppressing excess neuronal excitability throughout disease development, starting at the very early pre-clinical phase of the disease up until the chronic phase, and this is irrelevant to pre-synaptic functions.

### Deletion of IKKβ from CNS and peripheral myeloid cells resulted enhanced late long-term synaptic plasticity (late-LTP) after EAE

**Fig. 8:**
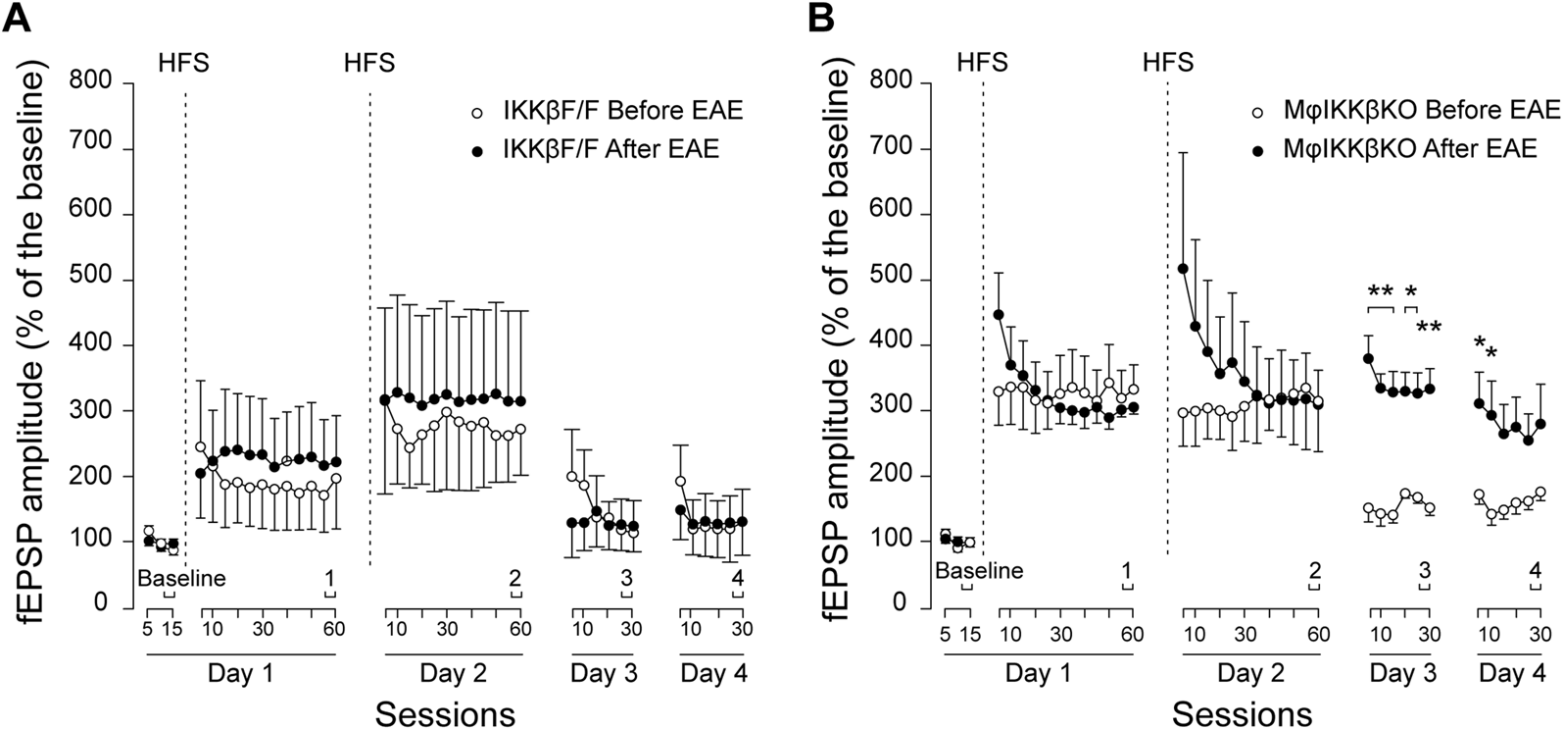
Global depletion of IKKβ from myeloid cells results in enhanced late-LTP. **A** Graph illustrating the time course of changes in fEPSP amplitudes (i.e., the LTP) after HFS of the Schaffer collaterals in IKKβF/F male mice before and at dpi 23 after MOG_35-55_-induced EAE, (*n =* 4). The HFS train was presented after 15 min of baseline recordings on 2 consecutive days, at the time indicated by the vertical dashed lines. The fEPSP amplitudes are expressed as a percentage of the baseline (Day 1, before HFS) amplitude (100%) in each group. Illustrated data were collected up to 60 min after HFS during the first (Day 1) and second (Day 2) days, and for 30 min on Days 3, 4, 5, and 6 after the first HFS. **B** Graph illustrating the time course of changes in fEPSP amplitudes (LTP) after HFS of the Schaffer collaterals in ΜφΙΚΚβΚΟ male mice before and at dpi 23 after MOG_35-55_-induced EAE, (*n =* 4). The HFS train was presented after 15 min of baseline recordings on 2 consecutive days, at the time indicated by the vertical dashed lines. The fEPSP amplitudes are expressed as a percentage of the baseline (Day 1, before HFS) amplitude (100%) in each group. Illustrated data were collected up to 60 min after HFS during the first (Day 1) and second (Day 2) days, and for 30 min on Days 3, 4, 5, and 6 after the first HFS. All mice in this graph were adult males, 3–5 months old. Data are shown as mean ± SEM and statistical analysis by two-way ANOVA, repeated measures, with Fisher LSD post hoc

We further compared the effect of deleting IKKβ from myeloid cells in long-term synaptic plasticity, a measure of postsynaptic function, before and after EAE induction. We induced LTP by high-frequency stimulation (HFS) of the hippocampal Schaffer collateral pathway and compared the evolution of fEPSPs evoked at the CA3–CA1 synapse in freely moving MφIKKβKO and IKKβF/F mice before immunizations and at dpi 25 after EAE induction. HFS was delivered as five trains (200 Hz, 100 ms) of pulses at a rate of 1/s and performed on days 1 and 2 using the same stimulation parameters and LTP was measured as the amplitude of fEPSPs evoked by each session of HFS followed by 1 h of post-HFS recordings. Subsequently, additional daily recordings of 30 min each were taken for up to day 4. Normalization of the results from the entire in vivo LTP protocol was performed using day 1 baseline recordings (15 min) for each condition. Before EAE the MφIKKβKO mice showed larger responses than IKKβF/F controls on day 1 and day 2, while on days 3 and 4 in both groups the fEPSP amplitude returned to baseline levels (Fig. 8A, B), which is in accordance with our previous study [7]. Comparison of LTP responses before and after EAE (at dpi 23) in IKKβF/F and MφIKKβKO mice revealed that in control group the fEPSP amplitudes in response to HFS were identical between the 2 conditions at all days after HFS (Fig. 8A). Similarly, in MφIKKβKO mice the fEPSP amplitudes were identical before and after EAE on days 1 and 2 following HFS, however at later timepoints, days 3 and 4, the amplitudes were significantly higher in the MφIKKβKO mice compared with controls (Fig. 8B). These results indicate that myeloid IKKβ is important for modulating LTP following HFS of Schaffer collaterals in EAE by regulating the excitability of neurons without affecting their ability to generate LTP.

Overall, these data show that depleting NF-κB activity in myeloid cells, both in CNS and periphery, enhances brain neuronal excitability and more specifically, affects neuronal postsynaptic functions such as the late-LTP, that also implies alterations in cognition. In relation to pathology, the homeostatic control of neuronal excitability by CNS macrophages through IKKβ seems to be highly important for delaying the onset of the disease, while at the chronic phase, deleting IKKβ from myeloid cells is protective against the motor clinical symptoms, but on the other hand, it negatively affects brain neuronal functions with direct consequence in cognitive abilities, such as learning and memory.

## Discussion

Our study provides novel insights on the cell-specific role of myeloid NF-κB activity in EAE and its cognitive comorbidities. We found that depleting IKKβ, the main activating kinase of the canonical NF-κB pathway, in CNS macrophages accelerates the onset of EAE, while depletion from peripheral macrophages reduces disease severity in the chronic phase. Of note, the suppressive action of inhibiting myeloid IKKβ to clinical EAE was paralleled by early brain neuronal hyperexcitability and enhancement of hippocampal long-term synaptic plasticity, which indicates alterations in cognition, such as in learning and memory.

The NF-κB is a ubiquitously expressed inflammatory transcription factor with multiple and cell-specific functions in health and disease [6]. In EAE, previous studies have shown that conditional depletion of IKKβ from total myeloid cells (CNS and peripheral macrophages) results in disease amelioration [32, 33]. This is in accordance with our findings that IKKβ depletion from myeloid cells is protective in chronic EAE, shown using mice with constitutive Cd11b-Cre driven IKKβF/F depletion (ΜφIKKβKO). Here, we show that ΜφIKKβKO mice further develop earlier clinical onset compared with controls, which was due to the action of IKKβ in CNS rather than in peripheral macrophages, as indicated by bone marrow chimera experiments. The protective action of CNS myeloid IKKβ was confirmed using a conditional genetic system, in which the IKKβ is depleted selectively from CNS macrophages using mice with inducible Cx3cr1-Cre driven IKKβF/F deletion with a previously described tamoxifen window system (MgIKKβΚΟ mice) [11, 28]. We found that these mice had significantly earlier clinical onset of EAE compared with controls, which was paralleled by earlier demyelination and faster immune cell infiltration in the spinal cord. They also showed exacerbated EAE and at the chronic phase of the disease, indicating that the ΙΚΚβ/NF-κB signaling pathway in CNS macrophages is protective, in contrast to peripheral myeloid IKKβ which has a disease promoting role at the chronic phase of the disease. A previous study has shown that conditional depletion of (TGF)-b-activated kinase 1 (TAK1), selectively from CNS macrophages in EAE causes less severe disease, diminishes CNS inflammation and subsequently decreases myelin damage in the spinal cord, by inhibiting the NF-κB, extracellular signal–regulated kinases (ERK)-1 and ERK2 (ERK1/2), and Jun N-terminal kinase (JNK) pathways [28]. The contradiction between these findings may reflect the role of TAK1 in multiple downstream pathways in neuroinflammation, compared to our system which specifically targets the canonical IKKβ–NF-κB pathway [9]. Furthermore, in another previous study it was shown that a depletion selectively from CNS macrophages of the tumor necrosis factor receptor 2 (TNFR2) resulted in earlier onset of EAE, with increased leukocyte infiltration and demyelination in the CNS, indicating that TNFR2-dependent signals in CNS macrophages suppress neuroinflammation [11]. Given that NF-κΒ signaling pathway can be induced via the activation of TNF cell surface receptor [34], these findings are consistent with our results that the CNS myeloid IKKβ/NF-κB signaling pathway is protective in EAE.

EAE is a model of MS that mimics autoimmune neuroinflammatory components of the disease, in which myelin-reactive T cells drive pathogenesis [3]. We found no evidence for effects of IKKβ deletion on the priming and differentiation of effector T cells in MφIKKβΚΟ or in MgIKKβΚΟ mice, even though myeloid cells participate as antigen-presenting cells in antigen-specific T cell activation and effector cell differentiation. Instead, our transcriptomic and immunohistochemistry data suggest that CNS intrinsic mechanisms are mostly affected in EAE in both mouse lines. Specifically, we show that inhibiting IKKβ from total myeloid cells in MφIKKβΚΟ mice, exerted protective functions in the chronic phase of EAE that was paralleled by increased expression of neuron-related genes, such as *Olig2* and *Snap25*, that are indicative of better CNS tissue recovery. On the other hand, depleting IKKβ specifically in CNS macrophages, using tamoxifen-inducible MgIKKβΚΟ mice, resulted in earlier clinical onset of EAE that was accompanied by alterations in gene networks related to neuronal functions during the pre-clinical stage of the disease. The functional effect of depleting myeloid IKKβ on neuronal activity was further measured by in vivo electrophysiology in the hippocampus of awake and freely moving MφIKKβΚΟ mice during MOG-induced EAE. We measured neuronal hyperexcitability in the CNS during the progression of disease in these mice, including pre-clinical, onset and chronic stages, compared to pre-immunization controls. We have previously shown in the same mice, that NF-κB signaling in CNS macrophages play a role in the healthy brain by exerting homeostatic regulation of neuronal excitability. Here we extend these findings to MS pathology, showing that CNS myeloid IKKβ is also important for regulating neuronal excitability in the context of autoimmunity and neuroinflammation. The neuronal component of the CNS during MS had been shown in previous studies [35], and brain neuronal hyperexcitability is believed to underlie the neurological comorbidities experienced by MS patients at different phases of the disease. Our finding that myeloid IKKβ is important for regulating neuronal excitability in EAE suggests that myeloid NF-κB activity might regulate cognitive function in MS and other neuroinflammatory diseases, and this is further supported by data discussed below. Furthermore, we cannot rule out the possibility that the NF-κB activity might be required by CNS macrophages to perform their homeostatic roles in controlling neuronal excitability in the healthy brain and in EAE. Our bulk RNA sequencing analysis on total RNA samples from whole spinal cord tissues showed no differences in the expression of homeostatic genes, such as *P2ry12, Tmem119, Thik1* [36], in MgIKKβΚΟ versus IKKβF/F control mice. However, such differences may be undetectable by the analysis we used, which was made on whole spinal cord tissue. Further cell-based RNA analysis or on RNAs isolated from pure CNS macrophagic populations will be informative to determine whether the NF-κB activity is required for the expression of homeostatic genes by CNS macrophages and therefore whether loss of CNS macrophagic homeostasis might be responsible for the neuronal disturbances caused by myeloid cell IKKβ deficiency.

Immunity in the CNS has generally been attributed to CNS parenchymal macrophages, whose origins and functions differ compared to peripheral myeloid cells [10]. Recent studies revealed that apart from microglia, other specialized types of macrophages also exist in the CNS, specifically at the CNS borders, including dural, leptomeningeal, perivascular and choroid plexus macrophages, collectively known as border associated macrophages (BAMs) [37]. Our study uses conditional and inducible Cre lines that differentiate the role of CNS versus peripheral macrophages in EAE but cannot discriminate the role of IKKβ in different CNS myeloid populations (parenchymal microglia and BAMs), a question that needs further investigation. We show, though, new evidence about the behavior of CNS parenchymal microglia in EAE in the absence of NF-κΒ activity. Microglia are a type of macrophage that resides in the CNS parenchyma and has a unique ramified morphology, with processes that are highly mobile, that constantly extend and retract to perform brain surveillance [38, 39]. This function is homeostatic with the aim to monitor, sense and tackle disturbances in the CNS tissue that may be caused by external factors, such as infections and trauma, or internal, such as neuronal death, accumulation of myelin debris and others. Their morphology is highly dynamic and change rapidly depending on the environmental stimuli and their activation status. We show that CNS parenchymal microglia, depleted for IKKβ change morphology at the very early time points of EAE, specifically at the pre-onset stage where peripheral lymphocytes are not yet infiltrating CNS tissue. One hypothesis for this early response could be that an EAE-induced transient BBB disruption at the pre-clinical stages of the disease, as has been described in previous studies [40], may cause infiltration of soluble immune mediators, such as chemokines, into the CNS parenchyma that activate the IKKβ deficient parenchymal microglia earlier than the controls. On the other hand, our data that neuronal gene markers are altered from the pre-clinical stages of EAE in mice with depleted IKKβ, and that this is associated with neuronal hyperexcitability during EAE progression also support a second hypothesis that depleting the ΙΚΚβ–NF-κB pathway from CNS macrophages renders these cells more susceptible to changes under inflammatory conditions, that could be a direct response to signals produced by neurons.

CNS parenchymal microglia–neuron communication has been described in several previous studies, in the healthy brain and in the context of disease, showing that neurons send on or off signals to microglia, depending on their activation status, with the second performing counterbalance activities to maintain tissue homeostasis. One such example is the interaction between neurons and microglia through the binding of the neuronal CD200 ligand to microglial receptors CD200R, that when inhibited, such as under inflammatory conditions, causes exaggerated microglial activation [41, 42]. Another example is the fractalkine receptor CX3CR1 and its ligand CX3CL1 that are expressed within the CNS by microglia and neurons, respectively [43]. Their interaction has also been implicated in neuroinflammation. Disruption of CX3CR1–CX3CL1 axis leads to microglial activation and hypersensitivity. Also, mice lacking CX3CR1 were more susceptible to EAE, with a significantly earlier onset and higher incidence of the disease [44, 45]. Control experiments in our study revealed that the earlier EAE observed in MgIKKβKO (full genotype: CX3CR1_Cre^+/−^_IKKβF/F) mice compared to IKKβF/F controls, was not due to the heterozygosity of the CX3CR1 but solely due to depletion of ΙΚΚβ in these mice (Additional file 5: Fig. S5C and SD). On the other hand, recent studies suggest that microglia dynamics are also able to regulate neuronal functions in pathological situations. Specifically, it was shown that inhibiting the Gi-dependent microglial dynamics led to sustained reduction of microglia brain surveillance and directed process motility that further caused induced spontaneous seizures and increased hypersynchrony upon physiologically evoked neuronal activity in awake adult mice. Also, in EAE, specifically before the onset of motor symptoms, it was shown that microglia in brain amygdala are more ramified and ‘less active’ and this might be related to the enhanced glutamatergic transmission that was also observed in this study, perhaps by performing less synaptic pruning, thus enabling more excitatory synapses to mature [46]. Our findings that IKKβ depleted CNS parenchymal microglia change morphology in the CNS of mice with EAE at the pre-clinical stage of the disease, and that this was accompanied by neuronal hyperexcitability, suggest that changes in microglia–neuron homeostatic communication act at the early time points of EAE in these mice and may be a cause for the earlier clinical onset. Whether IKKβ depleted CNS parenchymal microglia or neurons first get activated and send signals to the other at the early stages of EAE remains to be determined. As previously mentioned, we have shown that myeloid IKKβ activity is required for regulating neuronal excitability in the healthy brain [7]. We hypothesize that under inflammatory conditions, such as in EAE, neuronal excitability is dysregulated and has immediate effects on microglia, and therefore microglia–neuronal interactions, that ultimately have negative consequences for cognition.

MS is a progressive neuroinflammatory autoimmune disease of the CNS that is characterized by motor, sensory and cognitive decline, which may prevail independent of each other. Furthermore, learning and memory, information processing speed, attention and visual–spatial abilities are affected in 40–70% of MS patients [1]. Similarly, to that discussed above for neuronal excitability, we have previously shown that the NF-κB signaling in CNS macrophages play a role in the healthy brain by exerting homeostatic regulation and of long-term potentiation (LTP), a type of hippocampal synaptic plasticity known to be involved in learning and memory processes. Here, we found that the LTP evoked in vivo, in awake animals, by high-frequency stimulation of the hippocampal CA1–CA3 synapse is not affected in the brain of IKKβF/F control mice by MOG-induced EAE. However, knocking out myeloid IKKβ affected the late phase of LTP at the chronic phase of EAE (dpi 23). Regarding the effect of EAE induction on hippocampal LTP in control mice, our findings are in accordance with previous studies showing that LTP remains unaffected in C57BL/6 mice with EAE [1, 47]. However, other studies have showed that there is impaired hippocampal LTP in early EAE in C57BL/6 mice that is associated with differences in NDMA receptors and the action of the inhibitory GABA-ergic neurons [48, 49]. Differences in the experimental protocols used for inducing EAE and LTP in each study may be responsible for these contradictory observations. Our finding that depleting IKKβ from myeloid cells affects the late phase of hippocampal LTP when induced at the chronic EAE, suggests that cognitive abilities, such as learning and memory, may be altered at this stage of the disease in the ΜφIKKβKO mice, even though they show ameliorated clinical symptomatology compared with controls. We have previously shown changes in behavior, specifically in hippocampus-dependent associative learning, in these mice under healthy conditions [7], which support further the idea that caution is needed when using non-selective inhibitors of NF-κB activity to treat MS, as they might affect cognition of the patients as a side effect.

Previous studies, also reviewed by Zhou et al. 2020 [6], have shown that some of the approved clinical treatments for MS, such as dimethyl fumarate, FTY720 (fingolimod) and laquinimod have therapeutic effects by partially blocking the inflammatory cascade of the NF-κB pathway of the peripheral immune system or the CNS immune response mediated by astrocytes [6, 50]. Furthermore, in EAE, selective NF-κB inhibitors, such as pyrrolidine dithiocarbamate (PDTC), or peptides mimicking the NEMO-binding domain of IKK proteins NF-κB P65, have all shown to have beneficial effects by acting on peripheral immune responses. Likewise, the IKKβ-inhibitory compound PS-1145 has been shown to exert beneficial effects in EAE when administered during the induction and at the onset of the disease by suppressing the autoantigen-specific T cells responses [51]. Our data show amelioration of clinical EAE symptoms in mice with global depletion of myeloid IKKβ which is in accordance to these results, but we further show a protective role of CNS macrophagic IKKβ in early EAE, which was unknown so far. This beneficial effect of CNS macrophagic IKKβ in clinical EAE, together with the consequence of inhibiting total myeloid IKKβ in dysregulating synaptic plasticity emphasize the need of designing specific therapeutic molecules and protocols in the future that target pro-inflammatory actions of IKKβ in the peripheral immune system, while conserving neuronal regulation by CNS macrophages via NF-κB activity.

Collectively, we show that the IKKβ–NF-κΒ activity of myeloid cells have different roles in EAE depending on the cell type and the stage of the disease. IKKβ expressed in CNS macrophages is protective while that expressed in peripheral macrophages is disease-promoting and acts mainly at the chronic phase of the disease. Peripheral T cell effector functions were not affected but CNS resident mechanisms, such as microglial activation and neuronal hyperexcitability were observed at the very early stages of EAE, before the onset of motor symptoms, in mice depleted for myeloid IKKβ, suggesting that alterations in the homeostatic regulation of microglia–neuron communication at these early time points of EAE may drive the onset of the disease and the overall pathology in these mice. Lastly, depletion of myeloid IKKβ resulted in enhanced late long-term potentiation in EAE, suggesting that brain cognitive abilities may be affected by the use of non-specific therapeutic interventions that inhibit NF-κB activity in MS, which needs to be taken under consideration.

### Supplementary Information

**Additional file 1: Figure S1.** Detailed description of all EAE experiments done in MφΙΚΚβKO and IKKβF/F control mice. **A** Table showing the experimental parameters of the 4 individual EAE experiments that took place in MφIKKβKO and ΙΚΚβF/F control mice. **B** Representation of the mean clinical score of EAE (experiment 2 of table shown in **A**) for IKKβF/F and MφIKKβΚΟ female mice over 21 days post immunization with the peptide MOG_35-55_. **C** Representation of the mean clinical score of EAE (experiment 3 of table shown in **A**) for IKKβF/F and MφIKKβΚΟ female mice over 21 days post immunization with the peptide MOG_35-55_.

**Additional file 2: Figure S2.** MφIKKβKO mice show normal T cell priming and cytokine production in response to MOG. **A** T cell stimulation index, measured as the ratio between radioactivity counts of cells cultures in the presence of MOG_35-55_ peptide at the indicated concentrations and cells cultured with medium alone. The cells are splenocytes isolated from IKKβF/F and ΜφIKKβKO mice, which were in vivo pre-treated with MOG for 9 days. **B** T cell stimulation index, measured as the ratio between radioactivity counts of cells cultures in the presence of OVA protein at the indicated concentrations and cells cultured with medium alone. The cells are splenocytes and cells from draining lymph nodes isolated from IKKβF/F and ΜφIKKβKO mice, which were in vivo pre-treated with OVA for 9 days. **C** Quantification of the proportion % of isolated splenocytes from IKKβF/F and ΜφIKKβKO mice (re-stimulated in vitro with MOG) that were positive for IL-17, IFNγ, IL-10, IL-4, TNF and Tregs. **D** Quantification of the proportion % of isolated lymph nodes from IKKβF/F and ΜφIKKβKO mice (re-stimulated in vitro with MOG) that were positive for IL-17, IFNγ, IL-10, IL-4, TNF and Tregs. Numbers of mice are annotated as scatter dots on the bars. All mice were adult females 2-4 months old.

**Additional file 3: Figure S3.** RT-PCR on spinal cords isolated from IKKβF/F and MφIKKβKO mice at dpi 23 of EAE. Levels of *iNOS*, *Il1b*, *Ym-1*, *Arg1*, *Olig2* and *Snap25* mRNA in whole spinal cords from naïve (untreated) IKKβF/F and ΜφIKKβΚΟ mice and mice with MOG_35-55_-induced EAE at dpi 23, relative to Gapdh, as measured by quantitative RT-PCR. Numbers of mice are annotated as scatter dots on the bars. All mice were adult females 2-4 months old.

**Additional file 4: Figure S4.** Gene targeting analysis of the tamoxifen-inducible MgIKKβKO system. **A** Representative flow cytometry dot plots of cells isolated from spleens, spinal cords and brains of Cx3cr1-Cre^ER_YFP+/−^Rosa^26tdTomato+/−^ mice that were labeled for the pan-macrophage marker CD11b. **B** Quantification of the recombination efficacy, measured as the percentage of EYFP^+^CD11b^+^ cells that were also positive for tdTomato, after 2 shots of tamoxifen in mice shown in **A**. **C** Quantification of the recombination efficacy, measured as the percentage of EYFP^+^CD11b^+^ cells that were also positive for tdTomato, after 4 shots of tamoxifen in mice shown in A. Numbers of mice are annotated as scatter dots on the bars. All mice were adult females 2-4 months old.

**Additional file 5: Figure S5.** Tamoxifen-inducible MgIKKβKO mice have reduced response to LPS and the worst EAE compared to controls. **A** Representative flow cytometry dot plots showing isolated peritoneal macrophages from tamoxifen (TAM)—inducible MgIKKβKO and IKKβF/F mice that were double positive for CD11b and TNF when left untreated (left column) or after 24 h treatment with LPS (right column). **B** Quantification of the proportion (%) of CD11b + cells that were also positive for TNF in cultures of peritoneal macrophages from IKKβF/F and MgΙΚΚβKO mice, both with and without TAM, that either left untreated or treated with LPS for 24 h. **C** Mean clinical score of EAE for IKKβF/F mice without (w/o) TAM and MgIKKβKO mice with (w) and without TAM (*n =* 7 mice per group) over 28 days post immunization with the peptide MOG_35-55_. Numbers of mice are annotated as scatter dots on the bars. All mice were adult females 2-4 months old.

**Additional file 6: Figure S6.** Depletion of IKKβ from CNS macrophages did not affect cuprizone-induced white matter pathology. **A** Specimen images of paraffin-embedded coronal brain sections from IKKβF/F and MgIKKβKO mice (pre-treated with 4 shots of tamoxifen) stained with Luxol fast blue (LFB) showing the spatiotemporal evolution of demyelination and remyelination in the corpus callosum in the cuprizone model. **B** Specimen images of paraffin-embedded coronal brain sections from IKKβF/F and MgIKKβKO mice immunolabeled for Iba-1 showing the spatiotemporal evolution of microgliosis in the corpus callosum in the cuprizone model. **C** Specimen images of paraffin embedded coronal brain sections from IKKβF/F and MgIKKβKO mice immunolabeled for GFAP showing the spatiotemporal evolution of astrogliosis in corpus callosum in the cuprizone model. **D** Semi-quantification of the mean demyelination level in corpus callosum in IKKβF/F and MgIKKβKO mice shown in (**A**). **E** Quantification of the mean Iba-1 immunoreactivity in corpus callosum of IKKβF/F and MgIKKβKO mice shown in (**B**). **F** Quantification of the mean GFAP immunoreactivity in corpus callosum IKKβF/F and MgIKKβKO mice shown in (**C**). Numbers of mice are annotated as scatter dots on the bars. All mice were males 10-16 weeks old.

**Additional file 7: Figure S7.** MφIKKβKO mice show normal T cell effector functions. **A** Mean clinical score for IKKβF/F and MφIKKβΚΟ female mice over 12 days post immunization with the peptide MOG_35-55,_ which corresponds to the onset of the disease in the mφIKKβΚΟ group. **B** Quantification of the total number of infiltrating CD4 + T cells (left graph) and the proportion (%) of CD4 + positive cells that were also positive for IFNγ or IL-17 (right graph) in the spinal cord of MgIKKβKO and IKKβF/F mice with EAE shown in **A**. **C** Quantification of the proportion (%) of CD4 + positive cells that were also positive for IFNγ, IL-17, GM-CSF, TNFa or CD44^high^ in splenocytes isolated from MgIKKβKO and IKKβF/F mice at the onset of EAE shown in **A**. **D** Quantification of the proportion (%) of CD4 + positive cells that were also positive for IFNγ, IL-17, GM-CSF, TNFa or CD44^high^ in lymph nodes isolated from MgIKKβKO and IKKβF/F mice at the onset of EAE shown in **A**.

**Additional file 8: Figure S8.** Microglia are hypertrophic in MgIKKβKO mice with EAE in the absence of CNS immune infiltration. **A** Representative confocal images of individual microglia from cryostat spinal cord slices of naïve and dpi8 of MOG-induced EAE MgIKKβKO and IKKβF/F mice, immunolabeled for Iba-1. **B** Quantification of the hypertrophic index and the total length of microglia shown in **A**. **C** Specimen images acquired with confocal tile scanning of cryostat brain slices from MgIKKβKO and IKKβF/F mice at dpi8 of MOG-induced EAE immunolabeled for CD45 (green) and Iba-1 (red) and stained with DAPI (blue).

**Additional file 9: Figure S9.** Short-term plasticity is unaffected in MφIKKβKO mice. **A**, **B** Paired-pulse facilitation (PPF) was evoked in control IKKβF/F (**A**) and MφIKKβKO (**B**) mice by stimulating Schaffer collaterals with a fixed current (30-40% of the amount required to evoke a saturating response). Averaged (5 times) fEPSPs paired traces were collected at interstimulus intervals of 10, 20, 40, 100, 200, and 500 ms. Data shown are mean ± SEM amplitudes of the second fEPSP expressed as the percentage of the first [(second/first) × 100] for each of the six inter-stimulus intervals used in this test (PP ratio). Pre-immunization: *n =* 5 mice per group; 6 dpi: *n =* 6 IKKβF/F and 5 MφIKKβKO; 12 dpi: *n =* 4 mice per group. All mice were males 3–5 months old.

## Data Availability

All data generated or analyzed during this study are included in this published article (and its Additional files) and are also available by the corresponding author upon reasonable request. The RNS-seq data are also available at the Sequence Read Archive (SRA) under the accession number SUB13818706.
