# Supplementary figures and images for: IKKβ deletion from CNS macrophages increases neuronal excitability and accelerates the onset of EAE, while from peripheral macrophages reduces disease severity
Source: J Neuroinflammation. 2024 Jan 27;21:34. doi: 10.1186/s12974-024-03023-9 (PMC10821407; doi:10.1186/s12974-024-03023-9)

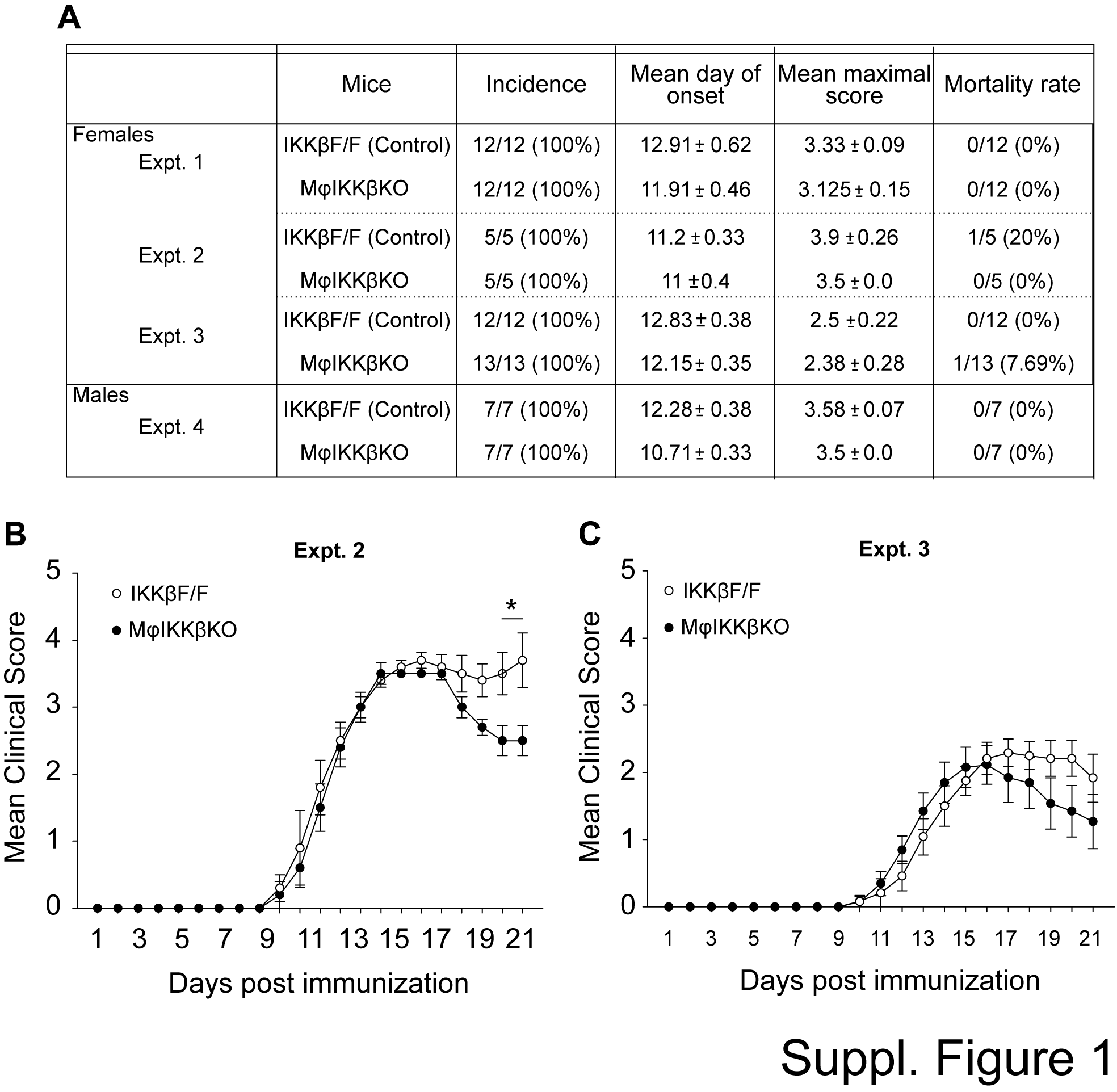

Supplement: Supplementary file 1 — Additional file 1: Figure S1. Detailed description of all EAE experiments done in MφΙΚΚβKO and IKKβF/F control mice. A Table showing the experimental parameters of the 4 individual EAE experiments that took place in MφIKKβKO and ΙΚΚβF/F control mice. B Representation of the mean clinical score of EAE (experiment 2 of table shown in A) for IKKβF/F and MφIKKβΚΟ female mice over 21 days post immunization with the peptide MOG35-55. C Representation of the mean clinical score of EAE (experiment 3 of table shown in A) for IKKβF/F and MφIKKβΚΟ female mice over 21 days post immunization with the peptide MOG35-55. [file 12974_2024_3023_MOESM1_ESM.tif]

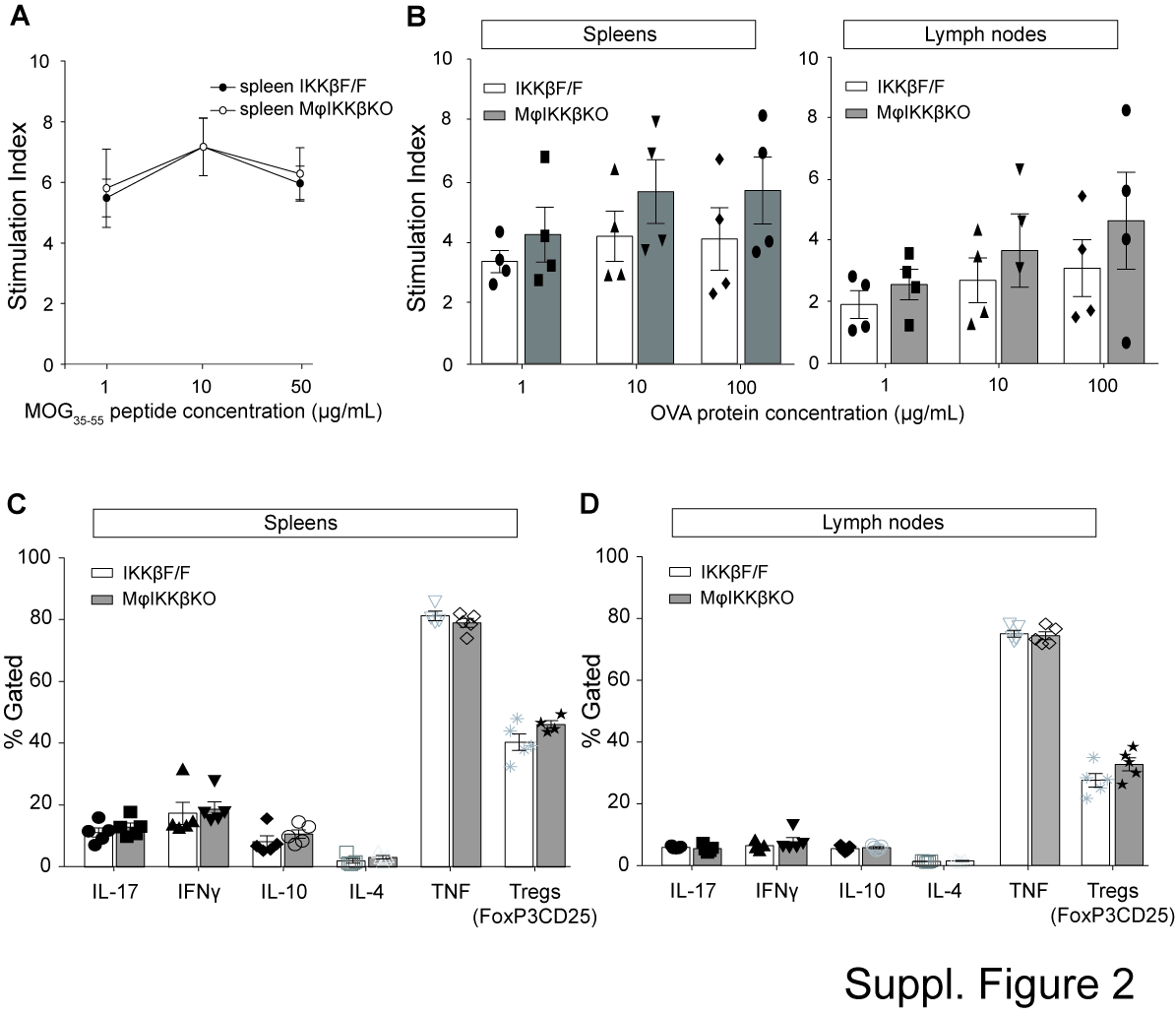

Supplement: Supplementary file 2 — Additional file 2: Figure S2. MφIKKβKO mice show normal T cell priming and cytokine production in response to MOG. A T cell stimulation index, measured as the ratio between radioactivity counts of cells cultures in the presence of MOG35-55 peptide at the indicated concentrations and cells cultured with medium alone. The cells are splenocytes isolated from IKKβF/F and ΜφIKKβKO mice, which were in vivo pre-treated with MOG for 9 days. B T cell stimulation index, measured as the ratio between radioactivity counts of cells cultures in the presence of OVA protein at the indicated concentrations and cells cultured with medium alone. The cells are splenocytes and cells from draining lymph nodes isolated from IKKβF/F and ΜφIKKβKO mice, which were in vivo pre-treated with OVA for 9 days. C Quantification of the proportion % of isolated splenocytes from IKKβF/F and ΜφIKKβKO mice (re-stimulated in vitro with MOG) that were positive for IL-17, IFNγ, IL-10, IL-4, TNF and Tregs. D Quantification of the proportion % of isolated lymph nodes from IKKβF/F and ΜφIKKβKO mice (re-stimulated in vitro with MOG) that were positive for IL-17, IFNγ, IL-10, IL-4, TNF and Tregs. Numbers of mice are annotated as scatter dots on the bars. All mice were adult females 2-4 months old. [file 12974_2024_3023_MOESM2_ESM.tif]

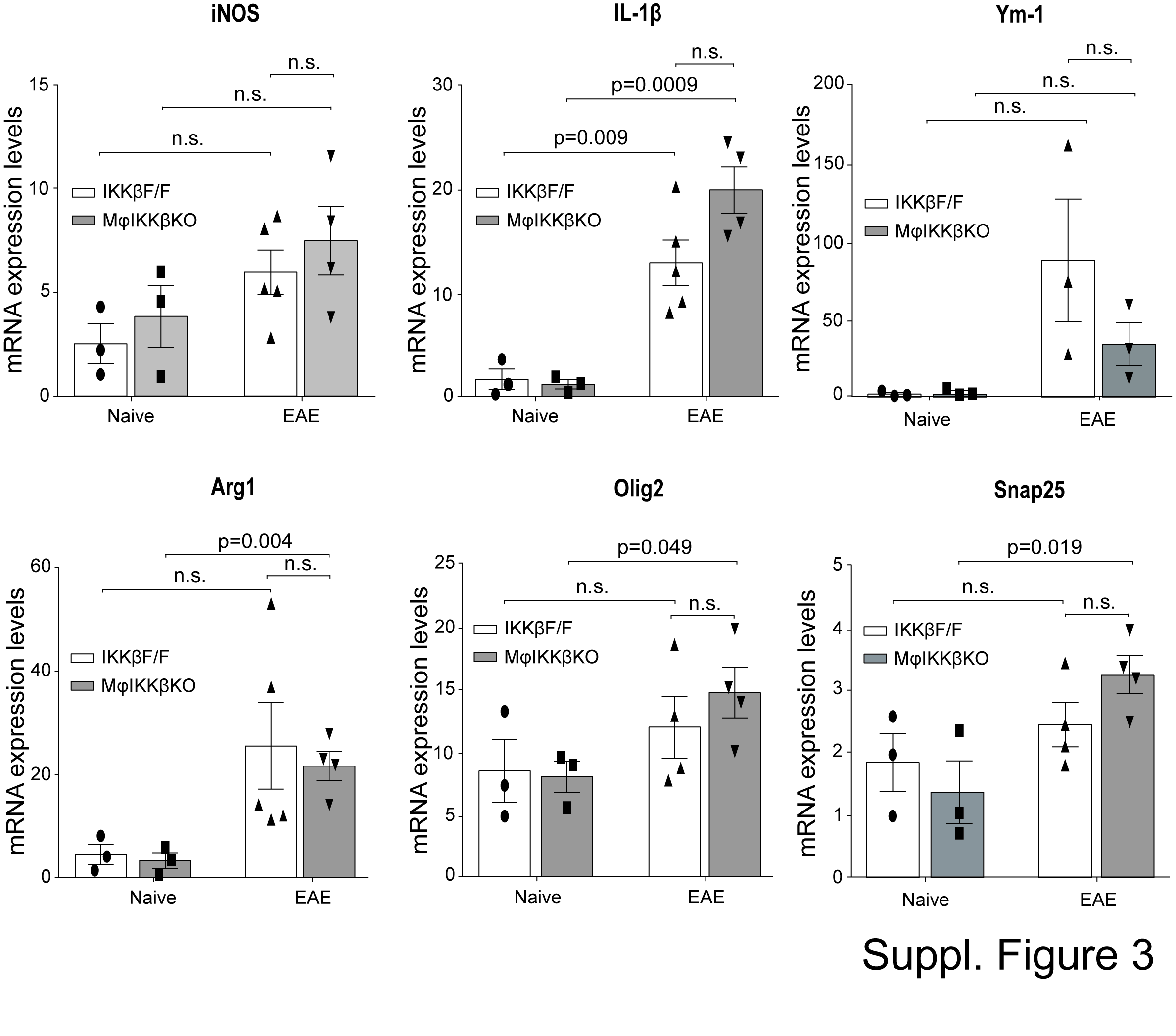

Supplement: Supplementary file 3 — Additional file 3: Figure S3. RT-PCR on spinal cords isolated from IKKβF/F and MφIKKβKO mice at dpi 23 of EAE. Levels of iNOS, Il1b, Ym-1, Arg1, Olig2 and Snap25 mRNA in whole spinal cords from naïve (untreated) IKKβF/F and ΜφIKKβΚΟ mice and mice with MOG35-55-induced EAE at dpi 23, relative to Gapdh, as measured by quantitative RT-PCR. Numbers of mice are annotated as scatter dots on the bars. All mice were adult females 2-4 months old. [file 12974_2024_3023_MOESM3_ESM.tif]

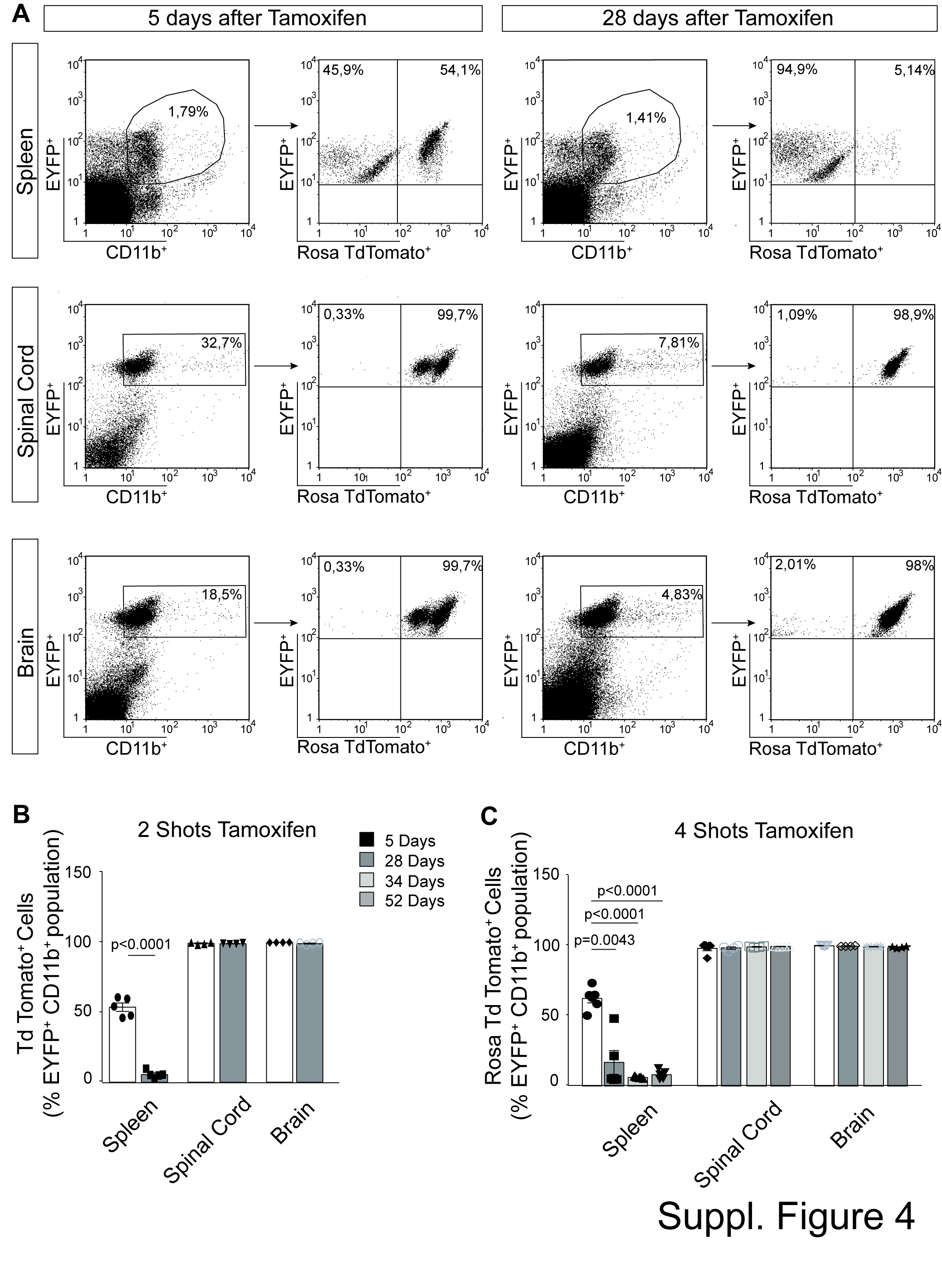

Supplement: Supplementary file 4 — Additional file 4: Figure S4. Gene targeting analysis of the tamoxifen-inducible MgIKKβKO system. A Representative flow cytometry dot plots of cells isolated from spleens, spinal cords and brains of Cx3cr1-CreER_YFP+/−Rosa26tdTomato+/− mice that were labeled for the pan-macrophage marker CD11b. B Quantification of the recombination efficacy, measured as the percentage of EYFP+CD11b+ cells that were also positive for tdTomato, after 2 shots of tamoxifen in mice shown in A. C Quantification of the recombination efficacy, measured as the percentage of EYFP+CD11b+ cells that were also positive for tdTomato, after 4 shots of tamoxifen in mice shown in A. Numbers of mice are annotated as scatter dots on the bars. All mice were adult females 2-4 months old. [file 12974_2024_3023_MOESM4_ESM.tif]

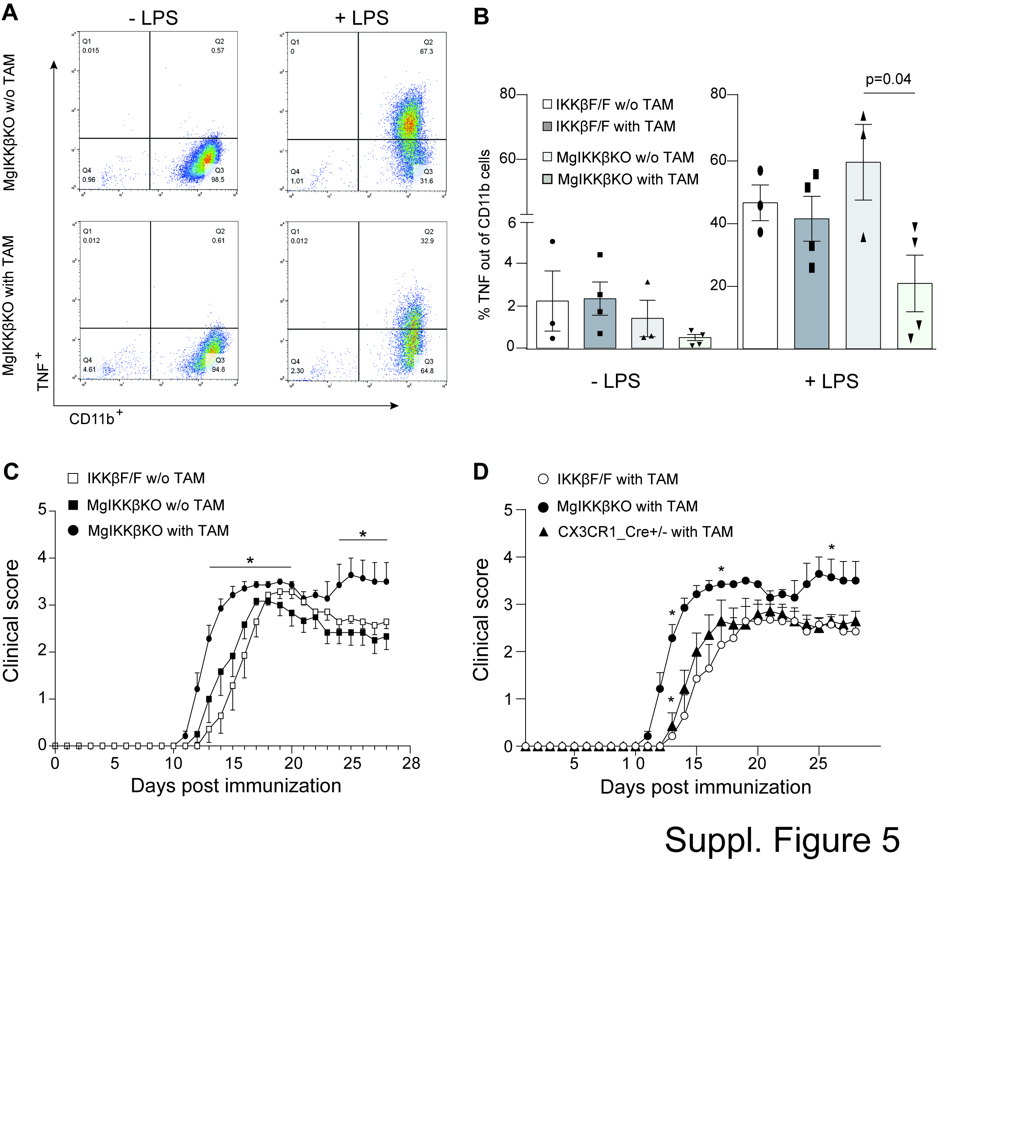

Supplement: Supplementary file 5 — Additional file 5: Figure S5. Tamoxifen-inducible MgIKKβKO mice have reduced response to LPS and the worst EAE compared to controls. A Representative flow cytometry dot plots showing isolated peritoneal macrophages from tamoxifen (TAM)—inducible MgIKKβKO and IKKβF/F mice that were double positive for CD11b and TNF when left untreated (left column) or after 24 h treatment with LPS (right column). B Quantification of the proportion (%) of CD11b + cells that were also positive for TNF in cultures of peritoneal macrophages from IKKβF/F and MgΙΚΚβKO mice, both with and without TAM, that either left untreated or treated with LPS for 24 h. C Mean clinical score of EAE for IKKβF/F mice without (w/o) TAM and MgIKKβKO mice with (w) and without TAM (n = 7 mice per group) over 28 days post immunization with the peptide MOG35-55. Numbers of mice are annotated as scatter dots on the bars. All mice were adult females 2-4 months old. [file 12974_2024_3023_MOESM5_ESM.tif]

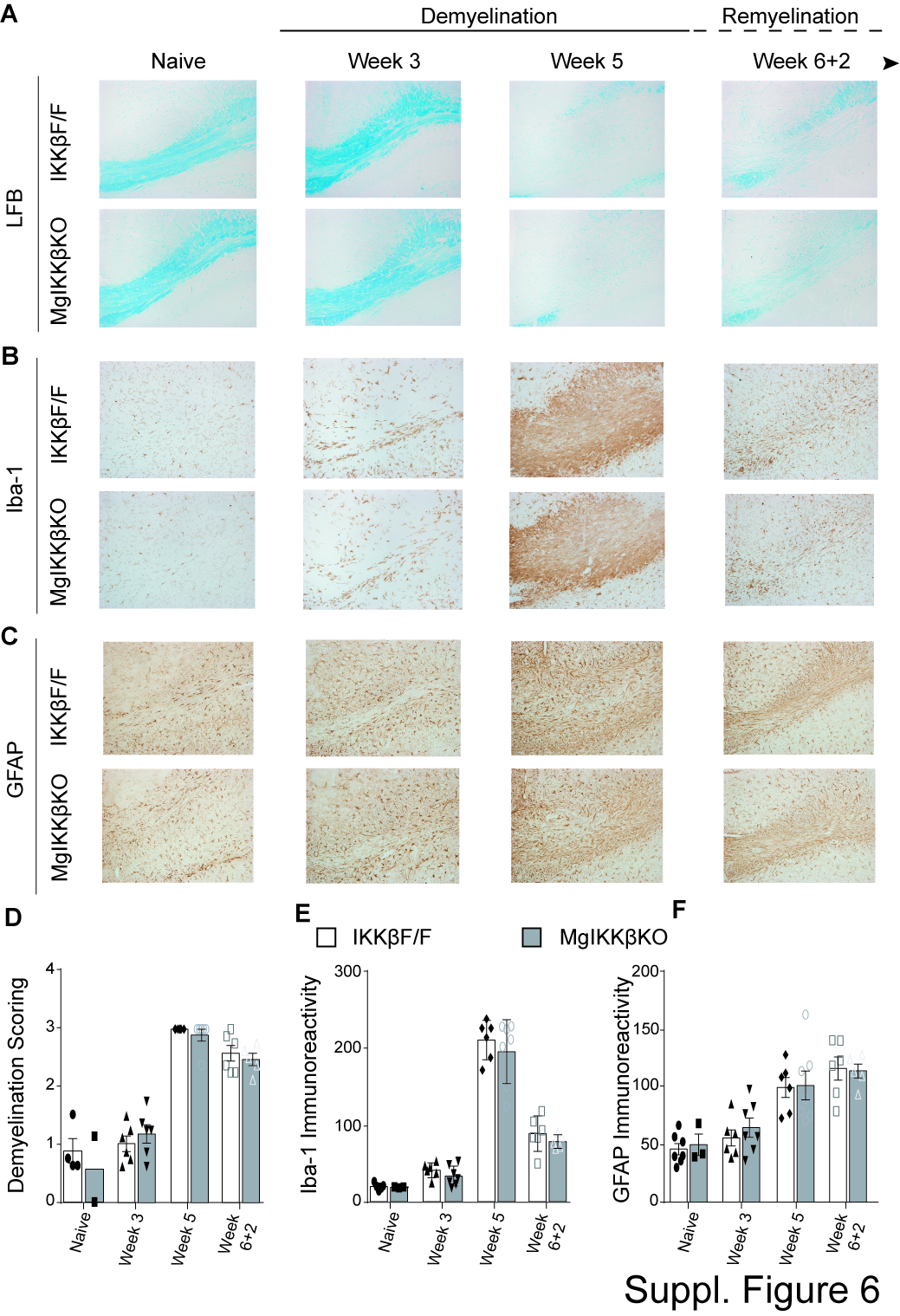

Supplement: Supplementary file 6 — Additional file 6: Figure S6. Depletion of IKKβ from CNS macrophages did not affect cuprizone-induced white matter pathology. A Specimen images of paraffin-embedded coronal brain sections from IKKβF/F and MgIKKβKO mice (pre-treated with 4 shots of tamoxifen) stained with Luxol fast blue (LFB) showing the spatiotemporal evolution of demyelination and remyelination in the corpus callosum in the cuprizone model. B Specimen images of paraffin-embedded coronal brain sections from IKKβF/F and MgIKKβKO mice immunolabeled for Iba-1 showing the spatiotemporal evolution of microgliosis in the corpus callosum in the cuprizone model. C Specimen images of paraffin embedded coronal brain sections from IKKβF/F and MgIKKβKO mice immunolabeled for GFAP showing the spatiotemporal evolution of astrogliosis in corpus callosum in the cuprizone model. D Semi-quantification of the mean demyelination level in corpus callosum in IKKβF/F and MgIKKβKO mice shown in (A). E Quantification of the mean Iba-1 immunoreactivity in corpus callosum of IKKβF/F and MgIKKβKO mice shown in (B). F Quantification of the mean GFAP immunoreactivity in corpus callosum IKKβF/F and MgIKKβKO mice shown in (C). Numbers of mice are annotated as scatter dots on the bars. All mice were males 10-16 weeks old. [file 12974_2024_3023_MOESM6_ESM.tif]

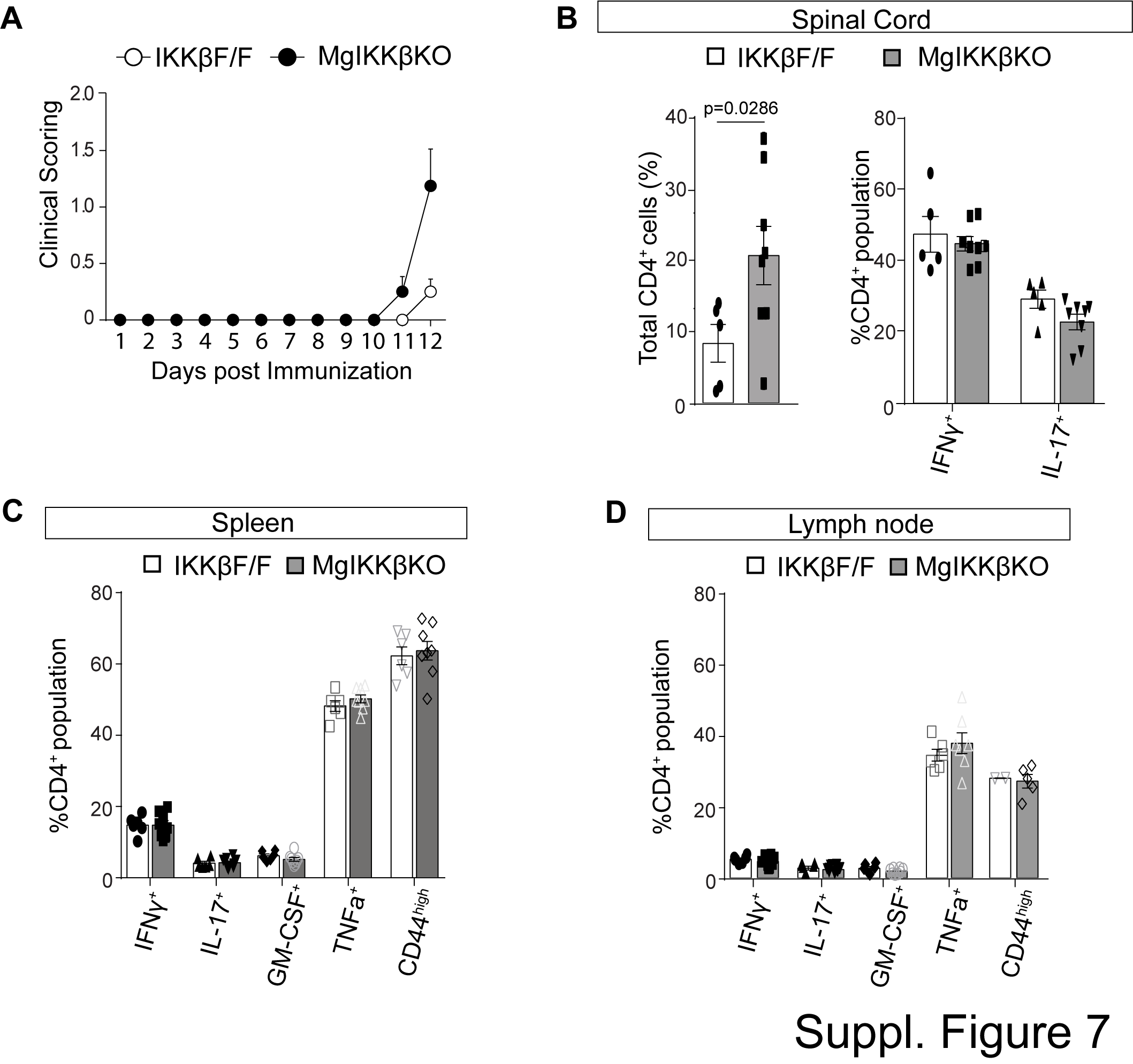

Supplement: Supplementary file 7 — Additional file 7: Figure S7. MφIKKβKO mice show normal T cell effector functions. A Mean clinical score for IKKβF/F and MφIKKβΚΟ female mice over 12 days post immunization with the peptide MOG35-55, which corresponds to the onset of the disease in the mφIKKβΚΟ group. B Quantification of the total number of infiltrating CD4 + T cells (left graph) and the proportion (%) of CD4 + positive cells that were also positive for IFNγ or IL-17 (right graph) in the spinal cord of MgIKKβKO and IKKβF/F mice with EAE shown in A. C Quantification of the proportion (%) of CD4 + positive cells that were also positive for IFNγ, IL-17, GM-CSF, TNFa or CD44high in splenocytes isolated from MgIKKβKO and IKKβF/F mice at the onset of EAE shown in A. D Quantification of the proportion (%) of CD4 + positive cells that were also positive for IFNγ, IL-17, GM-CSF, TNFa or CD44high in lymph nodes isolated from MgIKKβKO and IKKβF/F mice at the onset of EAE shown in A. [file 12974_2024_3023_MOESM7_ESM.tif]

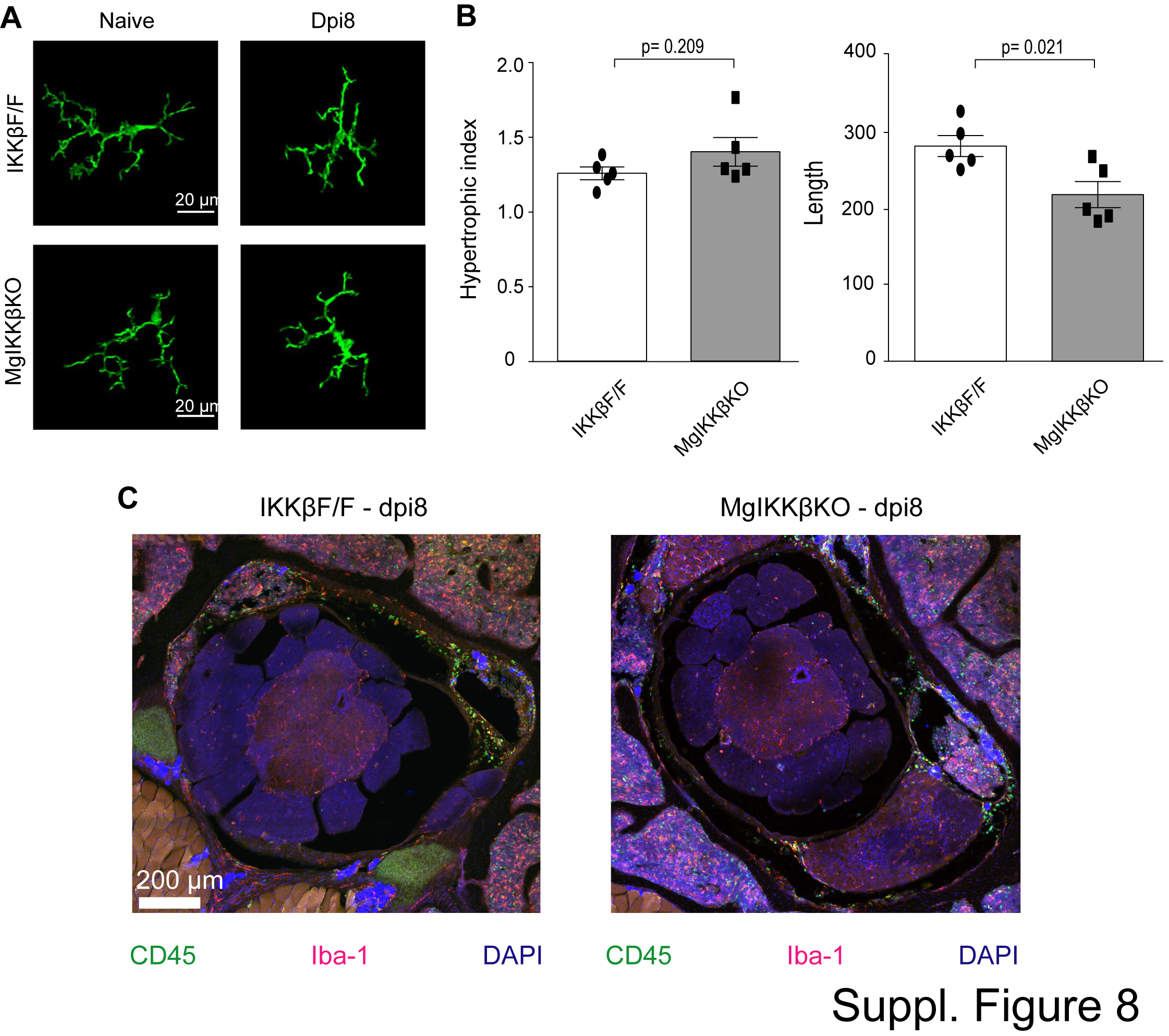

Supplement: Supplementary file 8 — Additional file 8: Figure S8. Microglia are hypertrophic in MgIKKβKO mice with EAE in the absence of CNS immune infiltration. A Representative confocal images of individual microglia from cryostat spinal cord slices of naïve and dpi8 of MOG-induced EAE MgIKKβKO and IKKβF/F mice, immunolabeled for Iba-1. B Quantification of the hypertrophic index and the total length of microglia shown in A. C Specimen images acquired with confocal tile scanning of cryostat brain slices from MgIKKβKO and IKKβF/F mice at dpi8 of MOG-induced EAE immunolabeled for CD45 (green) and Iba-1 (red) and stained with DAPI (blue). [file 12974_2024_3023_MOESM8_ESM.tif]

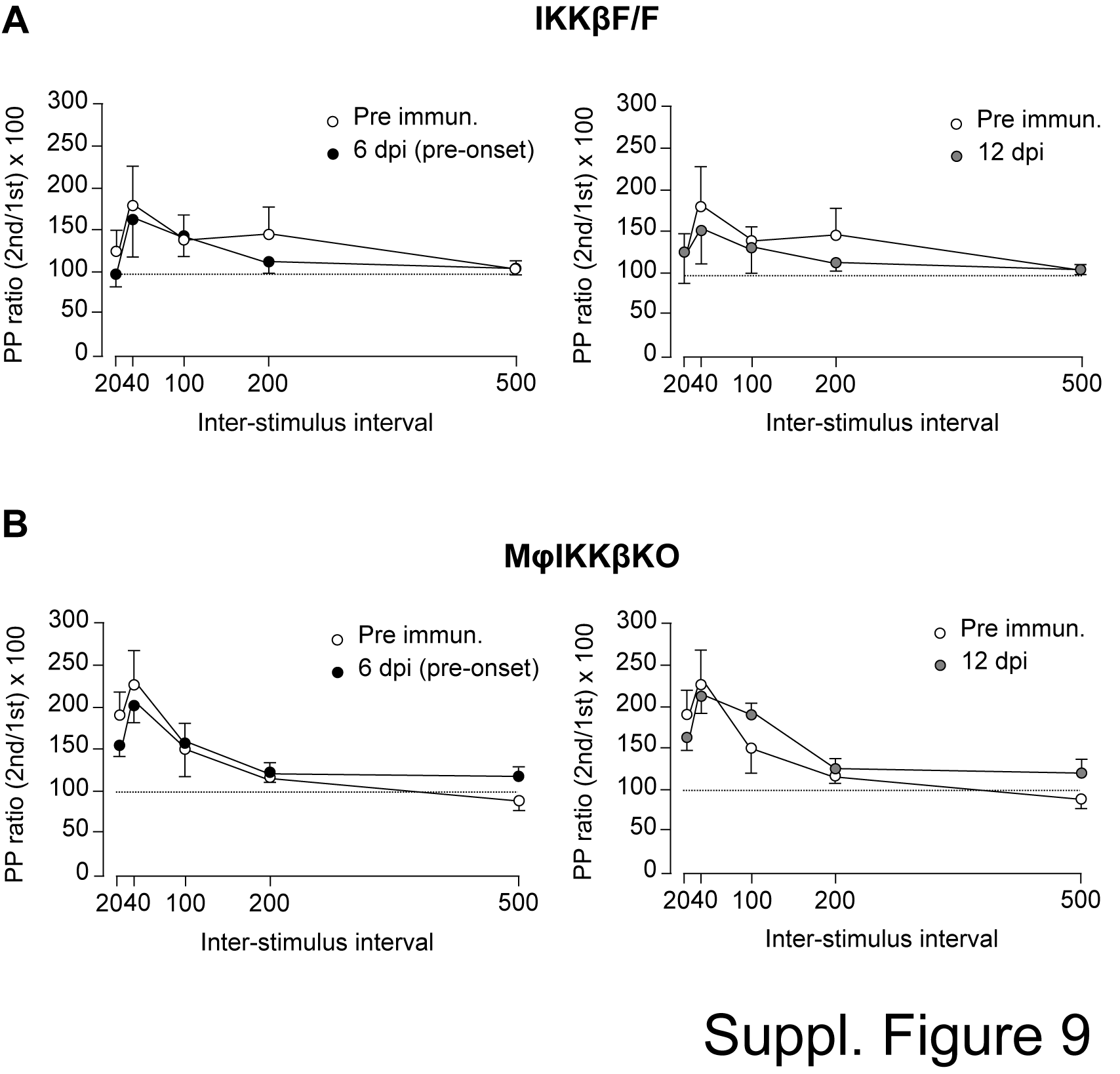

Supplement: Supplementary file 9 — Additional file 9: Figure S9. Short-term plasticity is unaffected in MφIKKβKO mice. A, B Paired-pulse facilitation (PPF) was evoked in control IKKβF/F (A) and MφIKKβKO (B) mice by stimulating Schaffer collaterals with a fixed current (30-40% of the amount required to evoke a saturating response). Averaged (5 times) fEPSPs paired traces were collected at interstimulus intervals of 10, 20, 40, 100, 200, and 500 ms. Data shown are mean ± SEM amplitudes of the second fEPSP expressed as the percentage of the first [(second/first) × 100] for each of the six inter-stimulus intervals used in this test (PP ratio). Pre-immunization: n = 5 mice per group; 6 dpi: n = 6 IKKβF/F and 5 MφIKKβKO; 12 dpi: n = 4 mice per group. All mice were males 3–5 months old. [file 12974_2024_3023_MOESM9_ESM.tif]
